# Nanotechnology development in surgical applications: recent trends and developments

**DOI:** 10.1186/s40001-023-01429-4

**Published:** 2023-11-24

**Authors:** Farzad Abaszadeh, Muhammad Hossein Ashoub, Ghazal Khajouie, Mahnaz Amiri

**Affiliations:** 1 Student Research Committee, Faculty of Allied Medicine, Kerman University of Medical Sciences, Kerman, Iran; 2 Department of Hematology and Medical Laboratory Sciences, Faculty of Allied Medicine, Kerman University of Medical Sciences, Kerman, Iran; 3 Cell Therapy and Regenerative Medicine Comprehensive Center, Kerman University of Medical Sciences, Kerman, Iran; 4 Neuroscience Research Center, Institute of Neuropharmacology, Kerman University of Medical Science, Kerman, Iran

**Keywords:** Nanotechnology, Tissue/organ engineering, Surgery, Implant material, Wound healing

## Abstract

This paper gives a detailed analysis of nanotechnology's rising involvement in numerous surgical fields. We investigate the use of nanotechnology in orthopedic surgery, neurosurgery, plastic surgery, surgical oncology, heart surgery, vascular surgery, ophthalmic surgery, thoracic surgery, and minimally invasive surgery. The paper details how nanotechnology helps with arthroplasty, chondrogenesis, tissue regeneration, wound healing, and more. It also discusses the employment of nanomaterials in implant surfaces, bone grafting, and breast implants, among other things. The article also explores various nanotechnology uses, including stem cell-incorporated nano scaffolds, nano-surgery, hemostasis, nerve healing, nanorobots, and diagnostic applications. The ethical and safety implications of using nanotechnology in surgery are also addressed. The future possibilities of nanotechnology are investigated, pointing to a possible route for improved patient outcomes. The essay finishes with a comment on nanotechnology's transformational influence in surgical applications and its promise for future breakthroughs.

**Graphical Abstract:**

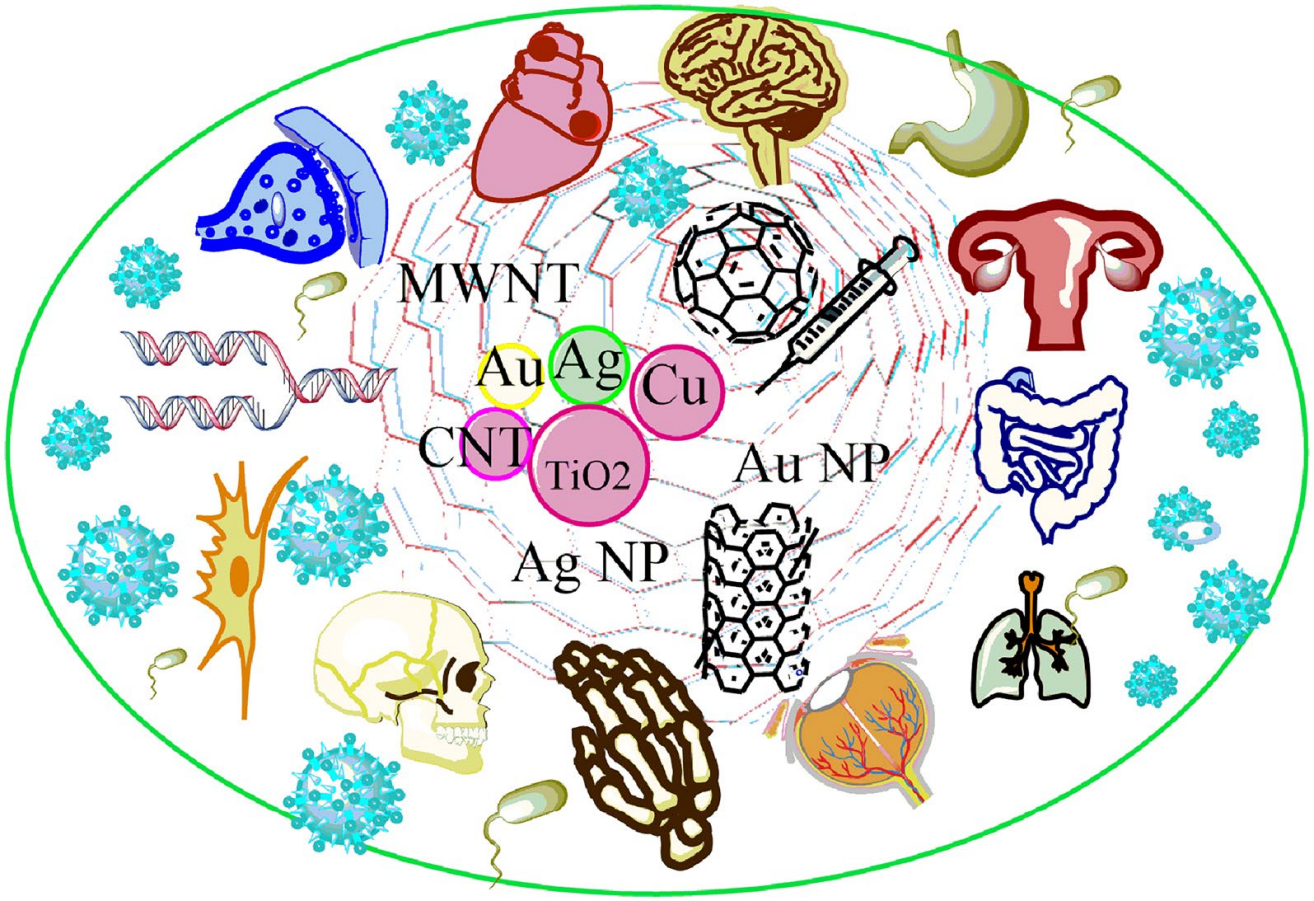

## Introduction

The operating room is one of the most stressful parts of the hospital, and nanotechnology has entered the operating room environment such as any other environment and is undergoing extensive changes [1, 2]. Due to their distinct size-dependent characteristics, nanostructures have recently attracted much attention. The capacity to create, manipulate, and utilize matter with nanometer-scale dimensions is known as nanotechnology. Because a material's dimensions significantly impact its characteristics at the nanoscale, particle size is crucial in nanotechnology [3, 4]. The design, manufacture, and use of innovative products that interact with biological, electrical, and chemical systems on the nanoscopic scale constitute nanotechnology. A subcategory of nanotechnology known as "nanomedicine" employs extremely targeted molecular manipulations to identify and cure-specific ailments. Richard Feynman, a physicist, initially presented the idea in the late 1950s [5]. He projected the capacity to manipulate matter at the nanoscale and indicated the prospect of controlling materials on the scale of individual atoms and molecules. The electronics industry, attempting to create tools for more compact electronic devices using silicon chips, made the first attempt at miniaturization [6, 7].

To build novel materials and gadgets with unique features, nanotechnology involves shaping individual atoms, molecules, molecular clusters, or surfaces into shapes. Nanotechnology aims to produce materials, tools, and systems with fundamental features and new functions [8–10]. The size of nanomaterials causes unique and different physicochemical properties of bulk materials or larger particles. Materials are more valuable and economical at the nanoscale than large, bulky materials. Nanoparticles have a higher surface area than volume, which increases the reaction. Nanotechnology is a promising field for producing new applications in medical health care and consumer products. The application of nanotechnology in medicine has increased in the last decade, and nanotechnology-based diagnostic and therapeutic methods are an excellent opportunity to accelerate the patient's healing process [11]. The biological activity of nanoparticles and their biological behavior has attracted many researchers, and an increasing number of nanoproducts are emerging to accelerate the patient's healing process. The use of nanotechnology in medicine, or "nanomedicine," affects the treatment of well-known disorders, diagnostics, and biological system control. Nanomedicine, also known as nanobiomedicine, uses nanotechnology in medicine. It influences biological system management and comprehension, illness detection, monitoring, and therapy. The application of nanotechnology in surgery has enormous promise [12–15]. As a smaller amount of stress is imposed upon a patient, scarring is reduced, and there are often fewer issues following the operation; surgeons continuously search for minimally invasive techniques to treat their patients. Humans cannot do precision surgery within cells, because their scalpels are millions of times more significant than a single cell. However, a surgeon operating a nanobot under computer control could [15–17]. Nanotechnology's most impacted diagnostic modalities include MRI, ultrasound, and nuclear imaging. They raise the standard of surgical diagnosis, planning, and execution. Scalpels and needles are the surgical devices most impacted by nanotechnology [18, 19].

Drug effectiveness is increased when explicitly delivered to the infection site, lowering morbidity and death in healthy neighboring tissues. In addition, this technique aids in lowering surgical trauma, which lowers the risk of postoperative problems [20]. Various nanotechnology applications include manufacturing equipment and implants, surgical sutures, dressings, ossification cases, infection control, instrument disinfection, modern drug delivery, nano-wound dressing, and tissue engineering products [15, 21–23]. This article has been reported to help design these cases and further applications of these materials. Nanoscience has entered this field extensively, and researchers have taken significant steps to overcome the limitations.

Nanotechnology is a promising field for producing new applications in the operating room [2]. The information obtained from this article can be valuable and useful for researchers to identify and accurately apply materials produced with nanotechnology in the operating room. Due to the length of this review and due to the familiarity of most readers with the concept of nanotechnology, this article does not discuss the concepts of nanotechnology, so for better understanding, they can refer to our previous papers [24–29].

## Nanotechnology applications in various surgeries

### Orthopedic surgery

**Fig. 1:**
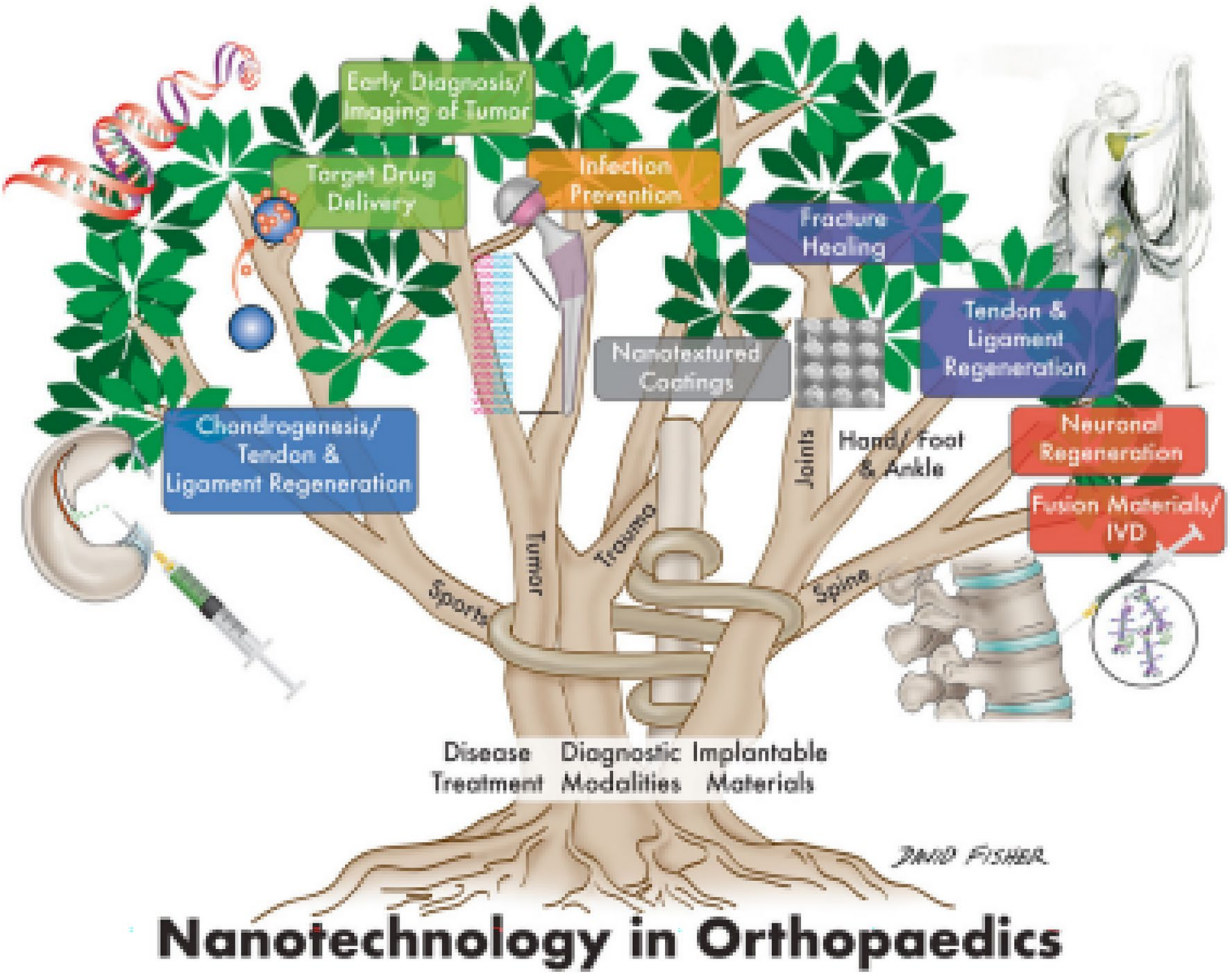
Diagram illustrating the areas and uses of nanotechnology in orthopedics [30]

Numerous novel tools are made available by nanotechnology for orthopedic applications (Fig. 1) [30]. The critical applications are osteochondral defects, meniscus repair and regeneration, osteochondral implant materials, and vertebral disks. It is crucial to deliver targeted drugs used to treat bone tumors. A significant factor in total joint replacement failure is the possibility of aseptic loosening (TJRs). Materials with nanotextured surfaces enhance osteoblast adhesion and osteointegration and are also helpful for treating bone defects. The human body's physical characteristics and nanoscale aspects promote cell development and tissue regeneration [31]. The use of implant coating materials, surface characterizing alterations, bone replacement materials, and tissue engineering techniques are some of the methods available to lower implant failure rates, prevent problems, and extend the life of the implants. Nanostructured implant coatings offer thermal insulation, preventing wear, corrosion, and erosion. The most popular coating materials are metalloceramic, hydroxyapatite, and nanostructured diamond [32, 33]. They are categorized as osteogenic growth factor, drug delivery coatings, metal ion coatings, peptides, and ECM components, titanium nanotube coatings, and bio ceramics made of calcium phosphate [34, 35]. Nanostructured diamond coatings (NSD and USND) are ultra-smooth nanostructured diamonds for titanium and cobalt-based alloy metal implant surfaces [36, 37]. The implants offer high adhesion qualities to titanium alloys and poor adherence to cobalt–chrome and steel substrates [38–40]. Orthopedic and dental implants frequently use nanostructured hydroxyapatite coatings [41, 42]. The implants are physically and functionally connected to the human bones via titanium and hydroxyapatite coatings. The electrophoretic deposition approach has recently been used to create the next generation of nanostructured hydroxyapatite coatings [43, 44]. Seven distinct types of diamond-like carbon (DLC) exist and share many of the same characteristics as the diamond [45]. Reduced abrasive wear of the implant surfaces is its essential characteristic. They are frequently utilized in implants for procedures to replace the hip and knee joints. Applications of nanomaterials in orthopedic surgery include extending the life of implants, treating osteoporotic vertebral fractures, preventing infections, treating orthopedic oncology, and using stem cells for regenerative medicine. Because of modifications to the physical characteristics and resulting energetics of the original materials, nanomaterials acquire improved physio-chemical properties. Reconstructive joint replacement, spinal implants, orthobiologics, and trauma are the primary implant uses [22, 46].

Orthopedic implants are frequently given to individuals with resections for bone malignancy. Standard materials, however, are not intended to stop the spread or return of cancer. Therefore, attempts are being made to create implants that promote healthy bone formation while halting the spread of cancer [47]. Selenium has been found to possess these qualities in the past, and nano-selenium implants have been shown to suppress the development of cancerous osteoblasts while boosting good bone function [46]. It was discovered that the selenium nanomaterial improved bone adhesion, calcium deposition, bone proliferation, and alkaline phosphatase activity more than untreated titanium implants. More recently, after being improved by grain refinement, nanostructured magnesium alloy implants showed anti-tumor capabilities. Unfortunately, less viable and sticky human osteosarcoma cells adhered to this substance [48, 49].

#### Implant material

**Fig. 2:**
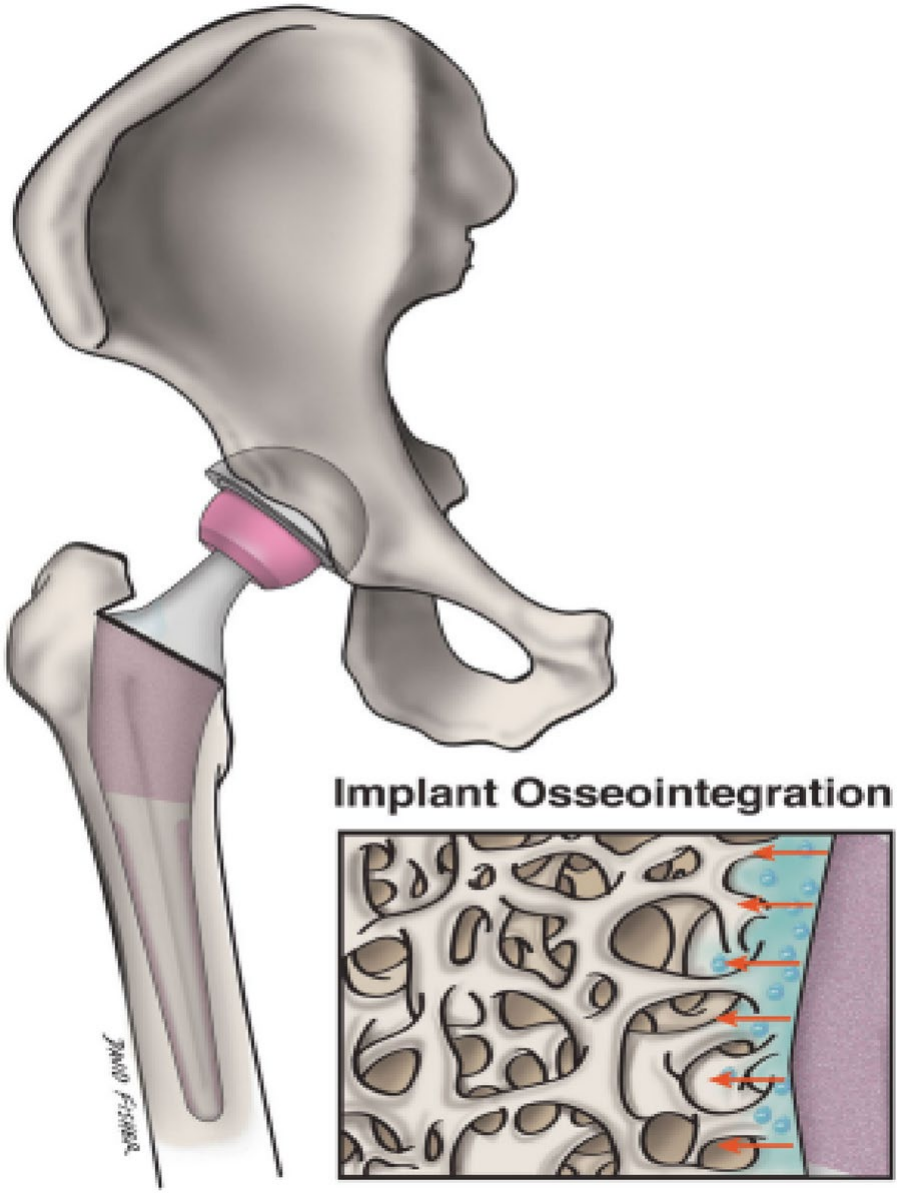
Nanostructured implants may promote implant osseointegration and surrounding osteogenesis to a greater extent than conventional implants, because they more closely resemble the environment of natural bone. In this illustration, a nanoengineered implant's surface is enlarged to illustrate how it interacts topographically with nearby bone [30]

Primary joint replacement surgery is quite successful. However, it has a short lifespan. The main goal of nanotechnology in arthroplasty is to create implantable materials that can work safely and effectively while increasing the average lifespan of implants and reducing infection. A more beneficial relationship between the implant and native bone can be created by altering the fundamental surface properties of the implant (Fig. 2) [30]. To improve implant osseointegration, osteoblast function and development have been enhanced using nanotextured implant surfaces. Notably, titanium implants' biocompatibility and mechanical qualities are improved by severe plastic deformation (SPD), which reduces metal's coarse grains into the nanoscale range by subjecting the metal to a complicated high-stress condition [50]. Due to worries about possible fracture, the use of ultra-high molecular weight polyethylene (UHMWPE) implants in arthroplasty has been restricted. However, there has been a growing interest in enhancing UHMWPE's mechanical strength using nanotechnology because of its superior biocompatibility characteristics and wear resistance [51]. A new composite made by mixing in carbon nanotubes has shown translational success and may 1 day be used as the lining of an acetabulum or as a part of the tibia. Modifying an implant's surface nanostructure can improve functioning, increase implant survival, and increase resistance to static and dynamic fatigue [52].

#### Cement

Nanotechnology is now employed to enhance widely used bone cement, such as polymethyl methacrylate (PMMA). It is standard practice to add antibiotics to bone cement, yet it is well-known that medicines frequently last for just a short time. When added to conventional cement materials, such as polymethyl methacrylate (PMMA), nanotechnology-based antibiotic transporters such as lipid nanoparticles, silica, and clay nanotubes may improve medication delivery and enable timed release. Antimicrobial characteristics of other non-antibiotic-based nanotechnology cement additives such as chitosan, silver, and dendrimer are also being studied. Furthermore, it is well-recognized that PMMA can trigger an inflammatory response that may result in implant failure through fibrous encapsulation and inflammation [53–56]. According to studies, adding nanostructured additives to PMMA led to higher levels of osseointegration and osteoblast activity. To enable X-ray visibility, ceramic particles such as zirconia and barium sulfate are frequently added to the cement. However, these particles have a detrimental effect on biocompatibility at the bone–implant interface. According to research by Gilliani et al. [57], these particles put into bone cement have been nanoscale modified, increasing their cytocompatibility and lowering their risk of mechanical failure. Together, these findings show the potential benefits of nanotechnology for enhancing bone cement effectiveness [57–59].

#### Chondrogenesis

**Fig. 3:**
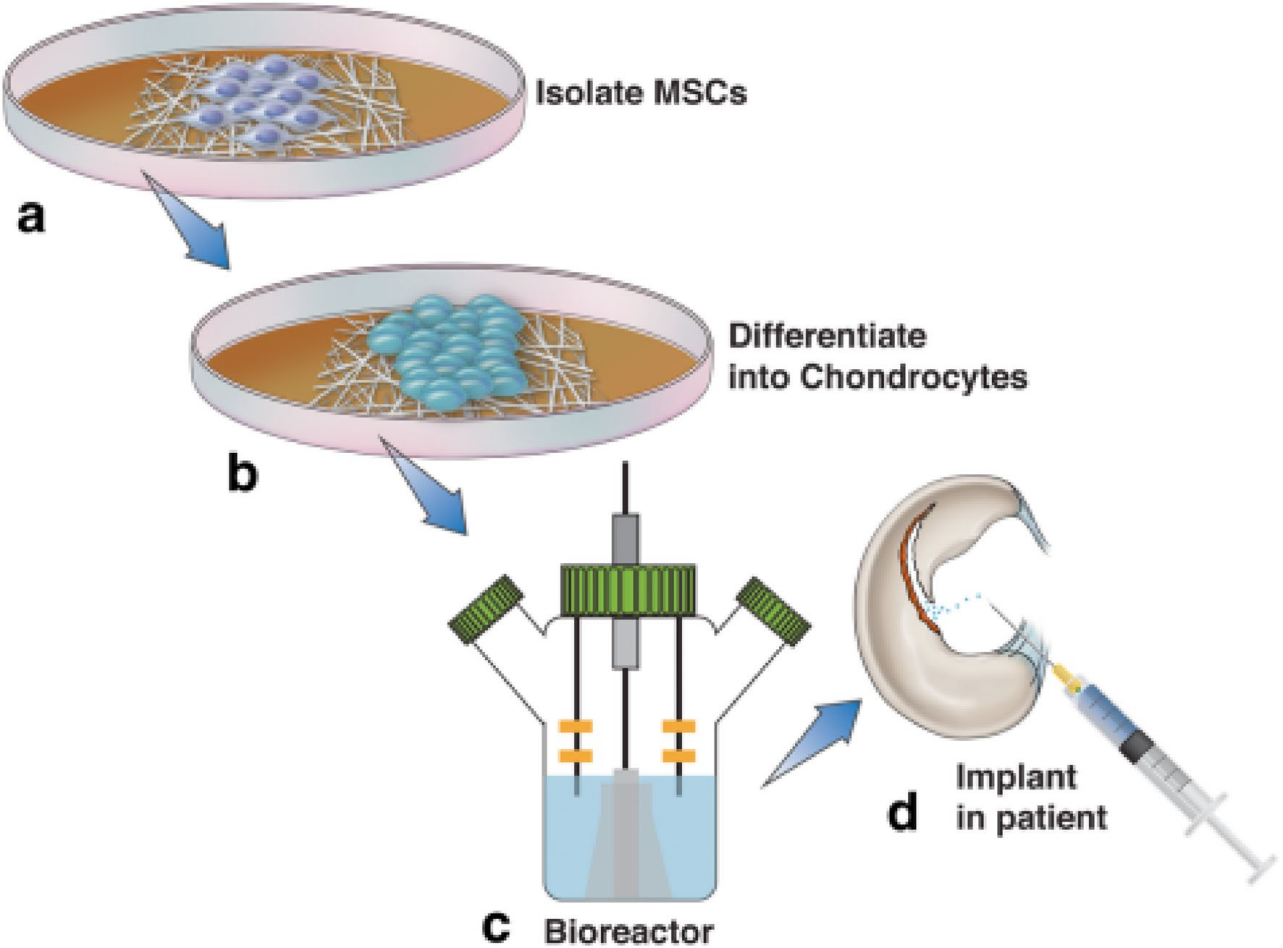
Although osteochondral abnormalities have been treated with limited success utilizing regenerative methods employing human MSCs, nanotechnology may make these treatments more effective. The normal flow of events for MSC therapy utilizing nanotechnology is shown in this diagram. The patient's MSCs are initially removed and cultivated in a growth medium (**a**). These cells are transplanted into the chosen scaffold material, developed into chondrocytes (**b**), grown in a bioreactor (**c**), and then reinserted back into the patient (**d**) [30]

The field of regenerative medicine has conducted substantial research on the problem of repairing cartilage deformities. Adult cartilage tissue lacks the necessary healing response for complete regeneration and, if ignored, will degenerate over time to develop osteoarthritis. Early progress has been made in preclinical attempts employing nanotechnology to supplement MSC treatment **(**Fig. 3) by creating a biocompatible scaffold that improves native cartilage healing [60–64]. Using pluripotent stem cells, Liu et al. [63] created a nanofibrous scaffold made of polycaprolactone and gelatin that improved articular cartilage repair and subchondral bone regeneration. According to Mahboudi et al. [60], MSC chondrogenic differentiation was significantly improved when a nanofiber-based polyethersulfone scaffold was used. In addition to the studies listed above, a broad range of alternative scaffolds, such as injectable hydrogels and materials based on peptides, are being researched to treat cartilage abnormalities. One pilot trial with 28 patients with osteochondral defects found that 70% of the lesions were fully filled 2 years after the transplant was placed. Other clinical trials have shown conflicting findings after a 3-year follow-up. More research is being done to determine the effectiveness and safety of these scaffolds. Although using nanoparticles as scaffolds for regenerative tissue engineering has been proven to alter cell adhesion, proliferation favorably, and phenotypic selection of chondrocytes, the application of nanotechnology in cartilage regeneration has not yet attained general clinical use [65–68].

#### Tendon healing

Despite recent advancements in surgical technique and post-operative care, adhesion development following tendon surgery remains a serious concern. An exciting alternative to enhance extrinsic and intrinsic tendon repair may be provided by developments in nanotechnology and medication delivery. Using hydrosol nanoparticles as drug carriers, Zhao et al. [69] created a method to enable the controlled release of mitomycin-C, a chemotherapeutic agent with the capacity to reduce post-operative adhesions. This made it possible to preserve mechanical strength equivalent to typically repaired tendons while reducing the establishment of tendon adhesion in vivo. Another development demonstrating the potential therapeutic utility of nanotechnology in tendon healing is tendon tissue engineering, specifically nanocomposite scaffolds. Several scaffolding materials are being researched. These scaffolds appear to meet the demands of regenerated tendons better than allografts, and studies have demonstrated that they increase healing and mechanical stability [69–72]. For example, a modified silk nano scaffold created by Sharif-Aghdam et al. [73] showed superior collagen content generation and viability. In addition, Huegel et al. [70] demonstrated that autologous nano scaffolds enhanced healing and mechanical stability in rat shoulders after supraspinatus repair surgery. Although clinical trials for tendon healing utilizing nanotechnology have not yet been conducted, several research investigations employing tissue engineering methods to mimic the bone–tendon interface are in progress.

#### Osteogenic properties of materials

Similar to arthroplasty, orthopedic trauma research using nanotechnology is focused on enhancing implant osseointegration and encouraging healthy bone formation after a fracture or non-union therapy. Surface alterations that allow for more accurate replication of the natural bone environment than traditional implants are the key to the potential effectiveness of nanostructured implants in trauma. Many research projects aim to design a bioactive scaffold for bone regeneration that would enable quicker healing and function recovery. Numerous studies have proven the osteogenic potential of these nanomaterials, and nanofiber scaffolds have been seen to promote cell migration and development during bone mending [74–77]. Although there has been much research into the nanostructuring of various materials, including metals, composites, polymers, and ceramics, it has not yet been applied in clinical settings due to unresolved concerns about clinical safety. By offering a viable alternative to bone allografting, and nanotechnology may also be able to assist in the management of nonunion deformities. Nanoengineered artificial bone grafts resembling natural bone structures have demonstrated pre-clinical effectiveness in increasing osteoblast adhesion and offering sufficient mechanical stability. An ultra-thin nanomaterial called nano silicates could also aid in the repair of bone deformities. When added to collagen-based hydrogels, they have shown good bone stiffness, porosity, and mineralization. The potential future use of nanomaterials in the therapeutic context is highlighted by their capacity to increase osteogenesis and improve the osseointegration of orthopedic implants [78–80].

#### Orthopedic infections

Orthopedic infection is still a serious issue, resulting in implant failure, delayed recovery, and further surgery. Infections are frequently caused by bacterial biofilms, which can only be effectively treated by removing the implant. Therefore, current efforts have concentrated on creating innovative nanoparticle-equipped anti-biofilm implants. For instance, a new vancomycin drug delivery system integrated into titanium femoral stems showed continuous release for up to 100 h. Over the past 10 years, orthopedics has developed a strong interest in nanophase silver, which is clinically employed in wound treatment [81, 82]. Anti-microbial nanophase silver dressings have been superior to standard dressings in preventing infections and promoting healing [83]. Compared to titanium implants that were not coated, germs were less likely to colonize covered titanium implants thanks to a silver nano powder coating created by Kose et al. [84]. Innovative research into IL-12 nanocoatings has shown promise in reducing infections caused by open fractures and may even alter immune responses to do so. Researchers recently created a titanium pedicle screw coated with silver nanoparticles, which has prevented biofilm growth on screws placed in rabbits. Overall, attempts to manage infection using nanotechnology have shown significant promise in preventing acute post-operative infections after trauma, spinal implants, and joint replacements [84–87].

#### Nanocoated or nano contoured implant surfaces

An implant is an artificial structure used to restore or stabilize bodily processes that have been destroyed. Many implants heal bone fractures and replace hip, knee, or temporomandibular joints. Problems with biocompatibility were addressed by introducing materials, such as titanium, cement, and polymers [88, 89]. The lifespan of conventional orthopedic/dental implants is constrained due to implant failure. Failure of implants can occur for various causes, including inadequate initial bone-integrating growth on the implant's surface, production of wear debris in the articulating parts of the implant, and imbalanced tension and strain on the surrounding tissues. If an implant is firmly placed into neighboring bone or implant material induces fast bone development, the failure rate may be reduced (osseointegration). Stainless steel and cobalt chrome alloys were initially the favored metallic implants for joint replacement due to their excellent mechanical qualities. However, due to these materials' high Young's moduli, stress shielding and bone resorption occurred. Since live bone needs to be subjected to some tensile pressure to remain healthy, stress-shielding should be avoided. At the tissue–implant contact, osseointegration reduces imbalances in stress and strain. Therefore, the mechanical properties of the surrounding bone tissue should match those of the implanted material [90–92].

Numerous methods have been explored to address these problems, such as manipulating the implant's surface, which would affect how osteoblasts work [93]. This is accomplished by supersaturating the implant's surface with calcium and phosphate [94]. Another strategy is to make the implant material's surface rougher [95]. Recent research has shown that the increased surface energetics of these proteins contribute significantly to their improved adsorption and confirmation on nanophase materials, which facilitate particular osteoblast adhesion. In addition, due to the wettability and surface properties of nanophase materials being near to protein size, they change the bioactivity of proteins [47]. In addition, surface characteristics, including implant or biomaterial composition, surface treatment, roughness, immobilization of different chemical agents to the surface, and nano features on the surface modify surface wettability and impact cell behavior. New nanoscale coatings will significantly improve surgical implants' fixation, biocompatibility, and wear properties. Like cells and tissues, nano-contoured implants or scaffold surfaces in regenerative medicine can significantly positively impact the development and proliferation of cells. The characteristics of various coating materials, including metalloceramic coatings, hydroxyapatite, and nanostructured diamond coatings, have been the subject of several studies. The most effective technique for creating nanostructured diamond coatings is chemical vapor deposition, which results in surfaces with a surface roughness of only 15 nm [92, 96, 97].

#### Bone replacement materials

NanOss™, a synthetic bone product, is made of hydroxyapatite nanocrystals, which have the strength of stainless steel and are scaled and formed, such as native bone crystals. VITOSS is an additional artificial substitute for cancellous bone transplants. Nanoparticles of -tricalcium phosphate with a diameter of around 100 nm are included in VITOSS®. Its porosity and structural design are created to imitate human cancellous bone [98, 99].

Compared to traditional tricalcium phosphate, higher porosity, and a greater surface area allow quicker and more efficient biosorption and vascular penetration. Artificial bone is a bone-like substance used in a lab for a bone transplant [100, 101]. The two main components of bone are hydroxyapatite and collagen fibers. Additionally found are keratan sulfate, lipid, and chondroitin sulfate. The material types prepared in bone grafting include minerals (hydroxyapatite) and organic polysaccharides (chitin, chitosan, alginate). Bone cement supplemented with nano clay fillers has improved mechanical qualities. Nanophase features may be seen in selenium, nanoceramics, alumina, titania, carbon, nanometals, cobalt chrome alloys, and nanocrystalline diamonds. To treat bone fractures, periprosthetic fractures during hip revision surgery, acetabular reconstruction, filling cages in spinal column surgery, osteotomies, and to repair bone abnormalities in children, bone replacement materials are employed [22, 47, 102, 103].

#### Bone grafting

The gold standard method for repairing skeletal abnormalities is bone transplantation, although there are drawbacks, including morbidity and resorption. Incorporating nanosurfaces into scaffolds may improve biocompatibility and lead to fewer issues [99, 104–107]. Nanotechnology to stimulate bone regeneration has been studied in recent years. In an experimental model, Tsukimura et al. examined the results of titanium alloy implants that had been both sandblasted and nanomodified. According to biomechanical analysis, the push-in forces for implants that had been sandblasted, alkali-treated, and heat-treated were all much higher than for implants that had merely been sandblasted. The histomorphometric study, which demonstrated increased bone-to-implant contact after implantation on the surface of the removed nanomodified implants, further supported these findings [108].

### Tissue regeneration in neurosurgery

**Fig. 4:**
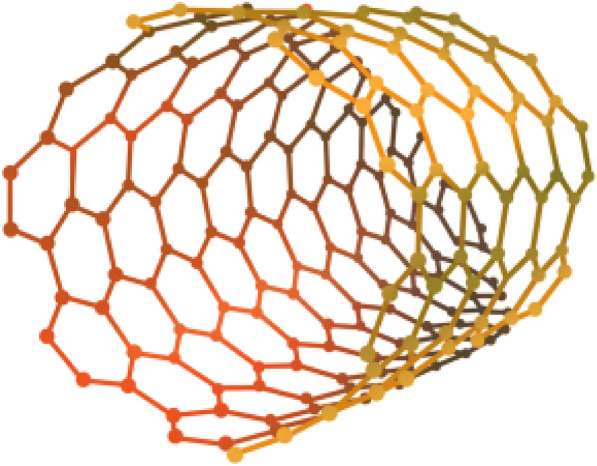
Carbon nanotube's fundamental structure is depicted in an illustration. These materials are highly sought-after in various orthopedic applications, including medication administration, implant scaffold fabrication, and nerve regeneration [30]

Loss of spinal mobility, degenerative post-discectomy spondylosis, and disc herniation recurrence are frequently side effects of surgical therapies for degenerative disc disease, such as discectomy and fusion. The need for nanotech research, including innovative cell-based therapies and tissue engineering for intervertebral disc (IVD) regeneration, has arisen due to inconsistent results and side effects with present treatments. These experimental treatments have shown that progenitor cells, including mesenchymal stem cells (MSCs), can differentiate into a phenotype resembling the nucleus pulposus [109–112]. Numerous studies have demonstrated that poly (-glutamic acid) nano complex injection treatment can improve the recovery of native IVD matrix. These nano complexes also have anti-inflammatory qualities in ex vivo animals. Growth factors are frequently used simultaneously to encourage differentiation and proliferation. Ineffective and expensive surgical treatments for peripheral nerve damage have already come under fire. By removing the morbidity connected with surgical operations to collect an autograft, neuron regeneration utilizing nanoengineering may offer an attractive option for treating peripheral nerve damage [109, 110]. In addition to providing higher mechanical flexibility than autografts, synthetic conduits made of carbon nanotubes and nano scaffolds may improve nerve regeneration through improved surface topographical interaction. It has been shown that carbon nanotubes may stimulate axonal development and even imitate some electrical characteristics of myelin (Fig. 4) [30, 113]. The development of nano scaffolds that closely match natural extracellular habitats has been made possible in large part by electrospinning methods [114, 115].

### Plastic surgeries

The development of diagnostic and therapeutic methods at the nanoscale has greatly helped plastic and reconstructive surgery. It includes craniofacial surgery, hand surgery, cancer/trauma/congenital repair, burns treatment, and aesthetic surgery. Plastic surgery is an enormously broad field. Advances in nanotechnology have a specific impact on medication delivery, tissue engineering, implant, prosthesis design, wound care, topical skin care, and regenerative medicine [116–119]. Everyday operations include plastic procedures. Breast implants used in mastectomy-related treatments for breast augmentation and reconstruction must be sturdy and robust enough to tolerate distortion. Nanoscale silicone rubber nanocomposite makes up the silicone breast prosthesis shell. To cure skin flaws, artificial skin is employed. The number of proteins required to repair neurons increases, and the time required for nerve regeneration decreases when type I collagen scaffolds are nanophase silver-impregnated [120, 121].

#### Tissue and organ engineering in plastic surgery

Skin defects have been treated using artificial skin. Advanced production facilities have improved aesthetic attractiveness with the availability of novel biomaterials and products that heal well. The quantity of adsorbed proteins needed for nerve repair is increased by Type I collagen scaffolds that are nanophase silver-impregnated [122, 123]. The daily practice of plastic surgery includes the use of implants and prosthetics. Breast implants used in mastectomy-related breast augmentation and reconstruction surgeries must be strong enough to endure deformation, capable of preventing the development of capsular contracture, and long-lasting [124]. The silicone breast implants silicone rubber nanocomposite shell is strengthened with nanoscale SiO_2_ [125]. Surface alterations by micro/macro texturization make the surface rougher. Available surface alterations include Biocell surface, patterned surface, and Siltex texturing. Silicone implants that have been coated in halofuginone, an anti-angiogenic substance, reduce the onset of capsular contracture [126]. Nanofiber coatings on the implants deliver chemotherapy medications tailored to the tumor's precise location at the tumor site [127]. Currently used systemic chemotherapy regimens do not have any unfavorable side effects. Maxillofacial surgery and dentistry might benefit significantly from using nanotechnology in the future, thanks to its applications in nanorobotics, nanomaterials, and biotechnology [128–130].

#### Materials to induce bone growth

To cure bone deformities, hydroxyapatite nanoparticles are employed. Protein-adsorbing nanopores on the modified surfaces of nanocrystals. Small voids left in tooth sockets following extraction are filled with calcium sulfate. It supplements the more durable bone graft material and is used to treat periodontal bone abnormalities [22, 47, 102, 103].

#### Breast implants

When new technology can be swiftly and readily taught and integrated into an established practice with little disturbance, plastic surgeons tend to pay attention to it. Surgeons are anticipated to make training investments and put up with schedule disruptions if a new technology would significantly improve their surgical practice and help them compete more effectively. New biomaterials with unique features that control cell functions have been produced due to advancements in nanotechnology, providing several therapeutic advantages. The use of breast implants in cosmetic and reconstructive breast surgery frequently results in various issues, including capsular contracture, double capsules, and late seromas [7, 17, 131, 132]. The goal of reducing those difficulties has led to the development and proposal of various breast implant surfaces. Different breast implant surfaces, a sterile, non-traumatic method, precise hemostasis, and local antibacterial medicines are currently used to treat the development of capsular contracture. Although a broad agreement has not yet been established, textured surfaces are thought to reduce the occurrence of capsular contracture. The most common causes of seroma include surgical site dead space, patient BMI, micro and macro repetitive stress, the use of acellular dermal matrix, and adjuvant radiation in patients undergoing reconstructive surgery [133–138]. By mimicking extracellular matrix topographical cues seen in the acellular dermal matrix (ADM) in synthetic implant surfaces, Kyle et al. [139] have described the production processes and attributes of breast implant surfaces based on nanotechnology. These authors claim this method may improve implant integration and function while lowering problems. In this work, a novel maskless 3D greyscale production method was used to reproduce ADM's micro- and nanoscale characteristics in polydimethylsiloxane (PDMS). Human-derived fibroblasts. Breast tissue cultures were performed on PDMS surfaces and contrasted with smooth and textured silicone implant surfaces that are readily accessible in the market. In comparison with commercially available implant surfaces, the scientists discovered that the PDMS with repeated ADM surfaces boosted cell adhesion, proliferation, and survival, as well as increased focal contact formation and disseminated fibroblast morphology. In addition, vinculin and collagen 1 were up-regulated, whereas IL8, TNFa, TGFb1, and HSP60 were down-regulated in fibroblasts on biomimetic surfaces. Similarly, Anderson et al. [140] investigated how nano topography affected cytokine production and cell shape. New functional materials could be developed as our understanding of cell interactions and organized nanoscale structures grows. These investigations highlighted the value of a unique strategy for creating functionalized biomimetic breast implant surfaces, which were shown to considerably lessen the in vitro acute foreign body response to silicone. Breast surgery may undergo significant modifications as a result of nanotechnology [126]. Chun and Webster [141] demonstrated that poor macrophage adherence and low protein absorption make nanostructured polytetrafluoroethylene (PTFE) less immunogenic in vivo. It has also been demonstrated that nanomaterials inhibit bacteria's capacity to form biofilms. Chronic biofilm-associated infection and antibiotic resistance are caused by bacterial adherence to surfaces [141, 142]. As a result, nanotechnology will offer crucial tools for creating a new class of substrates with antimicrobial qualities and for changing conventional surfaces to prevent bacterial attachment. The idea that such minute changes can have such a big impact is exciting, and the idea of nano surfaces has many applications in integrating breast implants and controlling the inflammatory response [142, 143]. These early findings also suggest that there are still a lot of unanswered concerns in the effort to integrate nanotechnology into clinical practice. The positive outcomes of in vivo studies can be regarded as a successful first step in introducing these implant surface modifications to plastic surgeons. However, conclusions regarding these new surface features' effect on implant performance for breast surgery applications will not be fully understood until long-term clinical studies are carried out [144].

#### Nanotechnology in wound healing

Nanotechnology is a fascinating new topic that has several uses in skin regeneration [145, 146]. Due to their structural resemblance to the extracellular matrix, nanofibers have attracted particular attention in the field of skin regeneration as scaffolds for skin regeneration; a wide range of polymeric nanofibers with unique characteristics have been created and studied. Nanofibrous materials can operate as delivery systems for medicines, proteins, growth factors, and other compounds in addition to supporting tissue healing. In addition, nanofibrous materials' shape, biodegradability, and other capabilities may be tailored to certain wound-healing circumstances. At various phases of wound healing, several nanostructured drug delivery systems have been employed to enhance healing, including nanoparticles, micelles, nanoemulsions, and liposomes. These nanoscale delivery systems have shown to have several advantages for the healing process of wounds, including decreased cytotoxicity of drugs, administration of poorly water-soluble drugs, enhanced skin penetration, controlled release properties, antimicrobial activity, protection of drugs against light, temperature, enzymes, or pH degradation, stimulation of fibroblast proliferation, and decreased inflammation [147, 148].

#### Soft tissue repair and healing

Clinical burn and trauma wound treatment have been greatly enhanced by nanotechnology [149]. Different dressing materials are made to promote wound healing at every stage of the healing process [150]. Nanofiber scaffolds in three dimensions mimic the natural extracellular matrix (ECM) and promote host tissue regeneration [151]. For the best wound healing and tissue recovery, mechanical integrity, fluid absorption, temperature management, and gas exchange qualities are crucial. Nanofibers offer all the ideal circumstances for an ideal healing environment [22]. Several studies show that chitosan is a physiologically active dressing that affects wound treatment positively. According to reports, applying chitosan to open wounds in dogs caused exudate, which has a high growth factor activity, as well as inflammatory cell infiltration and the creation of granulation tissue accompanied by angiogenesis. Although chitosan-membrane-based wound products have been studied in laboratory animals and humans, they are still in their infancy [152, 153]. It has been proven that chitosan microspheres have potent antibacterial action against S. aurous [154]. Due to its biocompatibility, biodegradability, hemostatic activity, anti-inflectional activity, and ability to hasten wound healing, chitosan was chosen as a dressing material [155]. N-acetyl glucosamine (NAG), a key component of dermal tissue and a prerequisite for healing scar tissue, is found in chitin and chitosan [156]. Its positive surface charge makes it possible to assist cell proliferation efficiently and encourages surface-induced thrombosis and blood coagulation. With the acidic groups of the cellular components of blood, free amino groups present on the chitosan membrane surface may form polyelectrolyte complexes [157, 158].

On the other hand, synthetic polymers are more affordable than biopolymer chitosan [159]; thus, replacing them with one might reduce the cost of chitosan-based films without compromising their functioning. One of the medicinal plants in the Liliaceae family is aloe vera. Since ancient times, it has been used as a laxative to cure many illnesses, including infections and dermatological diseases. The leaves of this plant are long, meaty, and thick and have twisted sides that finish with thorns. A long-chain polysaccharide of the acetylated glucomannan kind, together with other carbohydrates, makes up most of the "gel" found inside the leaf, composed of 99% water [160].

Along with avoiding infections, it is critical to consider the cellular activities that affect the healing process, such as cell migration and proliferation, inflammation, angiogenesis, and the production of collagen and other ECM elements [161]. Growth factors are crucial to these occurrences and determine whether the healing process is successful. Thus, several studies have created electrospun nanofibers for growth factor administration [147].

#### Wound closure

Researchers are looking for nano adhesives that take advantage of the delicate structure of biological surfaces. For example, geckos can scale vertical surfaces, whether dry, wet, or dusty [162]. They have no glue residue on their feet. Instead, they can achieve this because of tiny spatula-like protrusions on their footpads. Spatulas alone are nonpolar. The adhesion of nonpolar surfaces is caused by the brief creation of transient dipoles on nearby surfaces. The van der Waals attraction is based on these transitory dipoles. Minor and transient van der Waals attraction forces exist individually. They may be helpful in "scarless" surgery, in the treatment of tissues too delicate for suture, such as conjunctival, corneal, or lens tissues, in the treatment of photodamaged skin, or in the treatment of fragile tissues in patients who are immunosuppressed (such as patients taking prednisone) [163].

#### Wound and burn care

Two clinical care specialties that are currently gaining from advances in nanotechnology are wound and burn treatment [164]. New dressing materials have to be created to recognize the significance of a moist wound-healing environment based on the physiology and features of wounds. The health industry has looked into and introduced newer dressing materials based on an understanding of the biology of wounds, as opposed to the passive coverings previously used, which evolved from "natural" coverings, such as feathers, lint, grease, milk, wine, mud, leaves, and other strange concoctions. The objectives of an "ideal" wound covering are actively facilitated by modern wound dressings, which deliver local and systemic growth factors (e.g., cytokines), debride vitalized tissue and debris, act as a bactericidal and bacteriostatic agent, and maintain ideal moisture and pH balance of the wound milieu (e.g., by either removing excess fluid or providing extra oxygen) [165].

#### Therapy methods wound dressings

Wound dressings can now have customizable characteristics because of nanotechnology [166]. Wound dressings made with nanotechnology have behavior that can be customized for each individual wound and can stop chronic wounds from becoming worse. The release of antibacterial, anti-inflammatory, and drug/growth factors (GFs) may be adjusted in nanotechnology-based wound dressings in reaction to the wound environment [167]. Lipase and protease enzymes are two examples of the secreted virulence factors that are released by pathogenic bacteria that can cause tissue damage and cell death. Nanotechnology allows for the encapsulation of antimicrobial compounds in nanocontainers, where medication release is dependent on the presence of harmful microorganisms [168]. The optimal wound dressing should be mechanically stable enough, have a nanofiber-engineered structure, absorb wound exudate, moisturize the wound milieu, enable gas exchange, and release wound-healing mediators and antibiotics as needed. In this review, we concentrate on cutting-edge wound dressing technology that applies nanotechnology and regenerative medicine ideas. Different approaches to treating wounds include nanomicrobial-based dressings, antimicrobial, immunomodulatory nanoparticles, gene-altering/extinguishing technologies, and growth-releasing nanoparticles. A double layer of high-density polyethylene mesh with a silver coating surrounds the nanoparticles and nanofibers used in wound dressing. The three materials most frequently utilized in wound dressings are electrospun nanofibers, polyurethane, and silk fibroin nanofibers [120].

#### Nano biomaterials

A unique type of biomaterials known as nano biomaterials, which has at least one dimension in the nanoscale order, is an interface between biomaterials and nanotechnology [169]. Many biomaterials with short-term therapeutic uses are expected to be replaced in the following years by biodegradable biomaterials, which promote tissue regeneration and repair. For example, nanofibrous scaffolds have recently received much interest as skin healing scaffolds, mainly because they resemble ECM [170]. Synthetic medications, including antibiotics, anticancer medications, and analgesics, have been effectively mixed into nanofibers to create novel scaffolds and delivery systems. Even nucleic acids, peptides, and proteins are natural substances derived from plants [171]. It is possible to create nanofiber-based bioactive delivery systems using several techniques [172, 173]. The most basic of them is adding the bioactive material directly to the electrospun polymeric solution so it may be used when it is soluble. However, growth factors have been effectively encapsulated within the core of the fibers using core–shell nanofibers made by coaxial electrospinning and emulsion electrospinning, maintaining their bioactivity and continuous release. Post-treating electrospun fibers have increased the biocompatibility of the scaffold. Enzymes, adhesion molecules, and growth factors may all be immobilized to modify the surface properties of nanofibers and promote adherence and cell survival. Surface functionalization provides the benefit of modifying the surface features while keeping the mechanical qualities of the fibers and can be accomplished by plasma treatment, coating, or chemical techniques (such as hydrolysis and aminolysis). The tridimensional, highly porous structure of electrospun nanofibers acts as a barrier against microorganisms, preserving an adequate gas exchange and absorbing exudates when used as a wound dressing. Electrospun nanofibers can be loaded with a variety of bioactive compounds that can be delivered to the wound in a sustained manner. In addition, a variety of electrospun nanofiber composites with antibiotics have been created for use as wound dressings for the treatment of infected wounds. Along with avoiding infections, it is critical to consider the cellular activities that affect the healing process, such as cell migration and proliferation, inflammation, angiogenesis, and the production of collagen and other ECM elements [174–176]. Growth factors are crucial to these occurrences and determine whether the healing process is successful. Thus, several studies have created electrospun nanofibers for growth factor administration. An electrospinning technique may be used to create wound dressings made of synthetic and natural polymers, depending on the kind of wound. To create bioactive nanofibers, natural items such as plant extracts and essential oils can be added to the electrospinning polymer solution. In this regard, Sun et al. [177] mixed two organic substances with a biodegradable polyester electrospun wound dressing. The scientists integrated the ginsenoside (Rg3) from Panax ginseng into PLGA nanofibers, who then used pressure-driven permeation to coat the mat with chitosan. Chitosan increased the hydrophilicity of the mat and kept the ginsenoside Rg3 release constant for 40 days. Experiments in vivo showed that hypertrophic scar development and healing time were sped up. Nanofibrous materials have been thoroughly investigated as medication delivery systems and scaffolds for skin regeneration [178]. Other nanostructured drug delivery mechanisms, such as nanoparticles and liposomes, have been applied to enhance various stages of wound healing [179]. Numerous cutting-edge remedies powered by nanotechnology have been developed to address particular issues with the healing of chronic wounds. For the standardization of nanotechnologies, more study is still needed before these medicines may be used in the clinic. Skin wound healing is a highly coordinated, spatiotemporally controlled process that has been preserved throughout evolution. Hemostasis, inflammation, proliferation, and remodeling occur concurrently but in succession. Targeting the intricacy of the normal wound-healing process, cell-type specificity, an abundance of regulatory molecules, as well as the pathophysiology of chronic wounds is made possible by nanotechnology-based diagnostics and therapy techniques. In addition, there is a critical need for strong, more effective therapies for chronic wounds that may address a malfunctioning healing process at several cellular levels. These results highlight the requirement for creating and using innovative, nanotechnology-driven therapeutics [177, 180–182]. To effectively manage wound healing and reduce any potential complications that can arise during this process, nanotherapeutic techniques were developed using materials that have at least one dimension inside the nanoscale (1–100 nm), which are used in these materials. The adaptability and tunability of the nanomaterial's physicochemical features are its main benefit over its bulk equivalents (e.g., hydrophobicity, charge, size). Furthermore, nanostructures have distinctive characteristics due to the high surface area-to-volume ratio. Nanoparticles can, therefore, administer medicines in a prolonged and regulated manner, which speeds up the healing process. Nanoparticles with inherent qualities that help treat wounds and nanomaterials utilized as therapeutic agent delivery systems are the two primary kinds of nanomaterials used in wound healing [183–187].

#### Tissue engineering and instruments-based nanomaterials

The definition of tissue engineering is "the application of engineering and life science principles and methods toward a fundamental understanding of structure–function relationships in normal and pathological mammalian tissues and the development of biological substitutes to restore, maintain, or improve tissue function" [188, 189]. Three elements commonly combine to create tissue-engineered products: isolated cells, an extracellular matrix, and signal molecules, such as growth factors. New possibilities for the extracellular matrix, often known as the scaffold, are made possible by nanotechnology [190–193]. There are three primary purposes for the extracellular matrix. The first benefit is that it makes cell distribution and localization within the body easier. In addition, it creates and preserves a three-dimensional environment for the growth of new tissues with the proper structure. Third, it directs the growth of new tissues with suitable properties. The relationship between the cells and the extracellular matrix is crucial for the finished product's intended function [194]. Scaffold materials with micro- and nano-structured surfaces significantly positively impact cell adhesion and proliferation [195, 196]. To create three-dimensional constructions with the correct qualities, (nano) porosity control is crucial. In addition, the ability to adjust mechanical properties to resemble the target tissue closely increases the likelihood that tissue-engineered items will work as intended [197, 198]. The health care sector is developing extensively in tissue engineering and regenerative medicine [199].

**Fig. 5:**
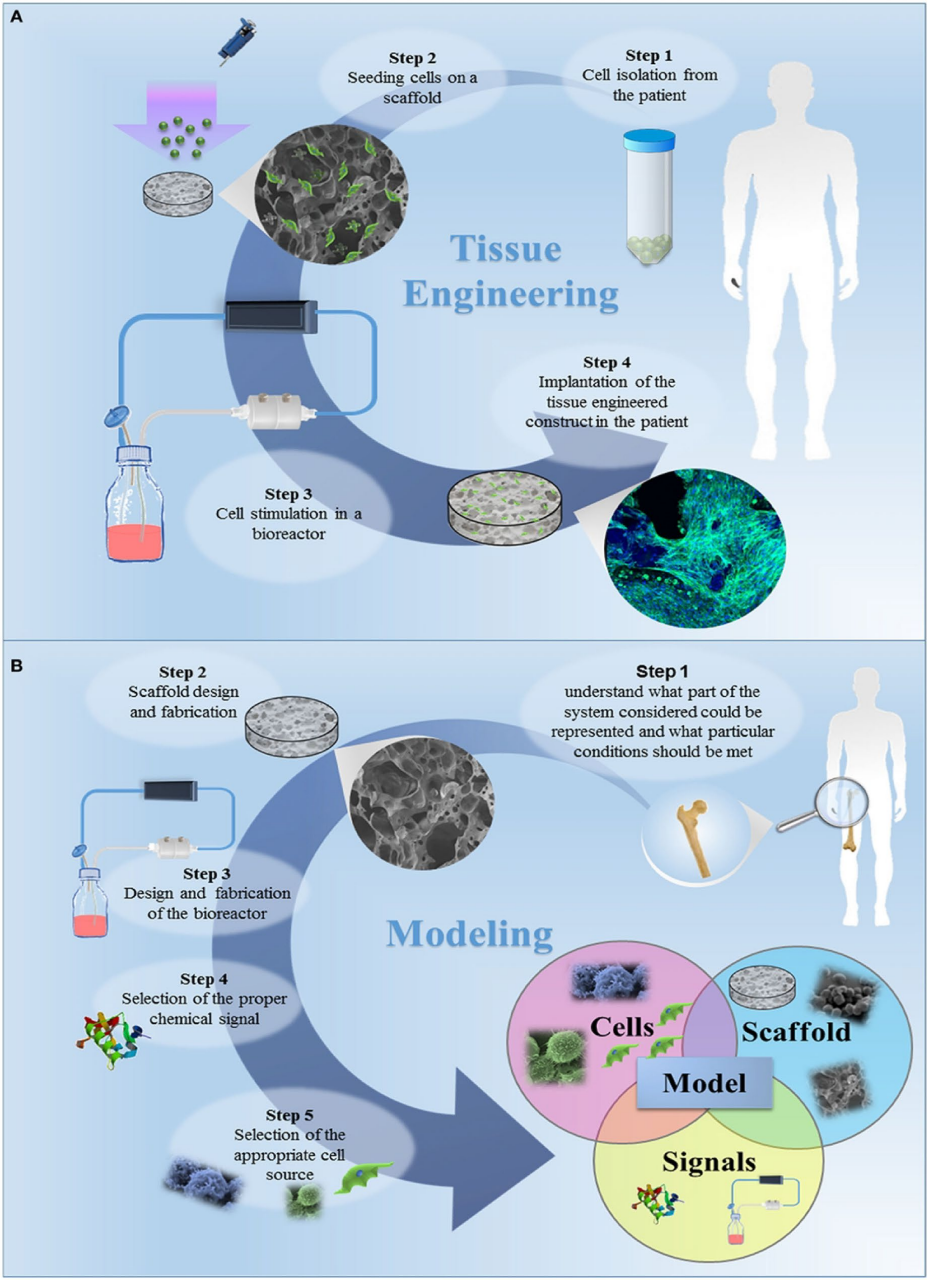
**A** Traditional tissue engineering (TE) paradigm involves isolating primary cells from patients and planting them on a three-dimensional (3D) porous scaffold particularly engineered to encourage cell proliferation and differentiation. The bioartificial system is created in a specialized setting, where a bioreactor provides metabolic, mechanical, and electrical stimuli. Cells begin to create an extracellular matrix in this tissue-specific engineered environment, leading to in vitro tissue development. The tissue-engineered product is subsequently implanted in the patient, where it undergoes an in-vivo remodeling process that facilitates tissue repair. **B** TE method for developing in vitro models [203]

Furthermore, practically all scientific and clinical research on structural fat grafting, which involves the transplantation of mesenchymal stem cells to treat congenital, oncological, traumatic, or cosmetic abnormalities, is carried out by plastic surgeons. By combining engineering ideas with life sciences, tissue engineering produces goods that enhance, restore, or sustain current biological systems (Fig. 5) [200–203]. Reconstructive plastic surgery uses cartilage engineering, which has long been used in orthopedic surgery. For example, it is a well-known approach to designing auricular cartilage for ear restoration [204, 205].

In addition, sophisticated nasal restoration following cancer, trauma, or congenital deformities is also being looked into using nasal cartilage. Skin defects have traditionally been treated using artificial skin. In recent developments, a scaffold made of polylactic and polyglycolic acids embedded with different growth factors is used, enhancing skin healing. Creating these goods can provide patients with a more natural-looking appearance following reconstruction in a safe, dependable, and repeatable manner using precise production processes and innovative biomaterials [206–208].

**Fig. 6:**
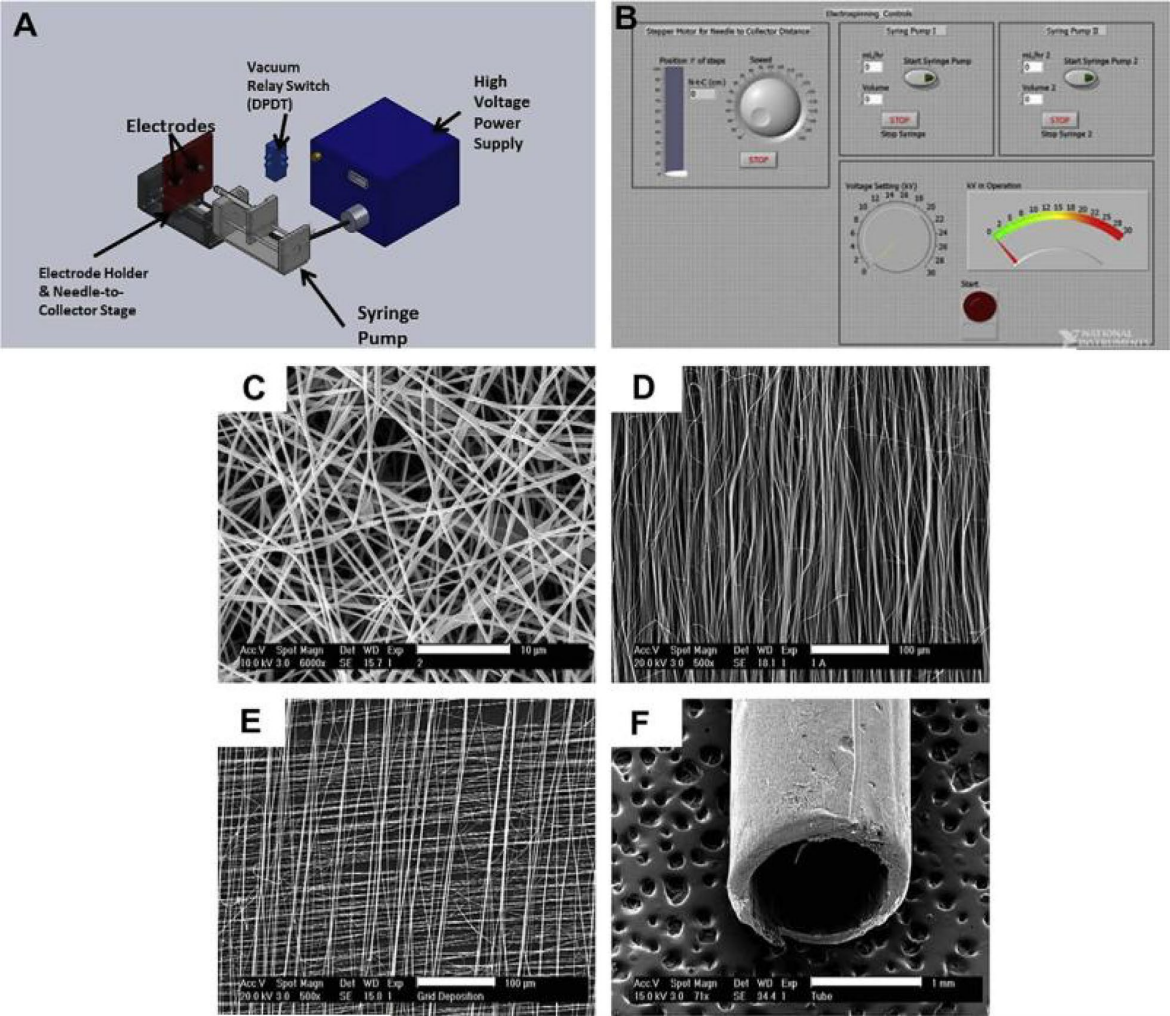
**A** Schematic illustration of our custom-built electrospinning device. Controlling the polarity of the collecting electrodes to regulate fiber orientation is possible using the vacuum relay switch (SEM images **C** through **E**). **B** Graphical user interface (GUI) is written in LabView 8.2 to control the construction parameters of electrospun scaffolds. **C** Typical scanning electron microscopy (SEM) picture of an electrospun scaffold with random fiber orientation (made from 10% gelatin B in HFIP). Scaffold with aligned fiber orientation (**D**), scaffold with gridded fiber alignment (**E**), and tubular scaffold built on a spinning mandrel (**F**) [209]

Engineering nano polymeric scaffolds to imitate the qualities of ECM, i.e., its fibrous nature and nanoscale features, is a crucial part of the current therapeutic drugs used in wound healing. Scaffolds are created using a variety of nanotechnology processes, including electrospinning, self-assembly, and phase separation (Fig. 6) [209]. The most popular technique for creating nanofibers is electrospinning. Electrospinning is a tried-and-true method for creating porous polymeric nanofibers, producing nano scaffolds with structural and physical characteristics similar to the ECM. PLGA/silk fibroin SF electrospun nanofibers were employed as hybrid scaffolds to encourage fibroblast adhesion and proliferation for better healing of diabetic wounds. Healing a Skin wound is a sequential and overlapping process that requires excellent spatial and temporal coordination. Proliferation, inflammation, remodeling, and hemostasis are all ongoing processes [184, 210, 211]. The wound healing method involves various cell types, such as fibroblasts, keratinocytes, immunological, endothelium, and progenitor cells. Growth factors, cytokines, and chemokines regulate several dynamic and ongoing molecular and cellular activities. The interplay between the skin and bacteria will determine how this process turns out. Cell-to-cell and cell-to-extracellular matrix (ECM) interactions are organized by the interleukin (IL) and growth factors signaling networks to heal a wound properly. Any changes to this order result in a persistent wound that never heals. The many methods for treating wounds include antimicrobial nano-based dressings, immunomodulating antimicrobial nanoparticles, gene-modifying/silencing technologies, and growth factor-releasing nanoparticles [182, 212].

#### Reconnection of injured tissues after surgery

Synthetic or biological tissue adhesives that rely on in situ polymerizations and crosslinking processes have arisen during the past several decades as supplementary approaches that can solve many problems related to sutures and staples, particularly in noninvasive procedures [213, 214]. However, there are certain drawbacks to the tissue adhesives currently on the market for clinical use, such as toxicity, significant swelling, inadequate strength, and a complex polymerization process [215, 216]. In addition, precise storage and preparation requirements must be met before using these polymer-based surgical materials in vivo. Recent developments in nanotechnology have impacted the area of tissue adhesives, either by providing novel functions (such as hemostatic or antibacterial qualities) or by enhancing their mechanical properties and adhesion force to neighboring tissues [217, 218]. To lower the risk of infections or bleeding after operations, nanoparticles with antibacterial and hemostatic characteristics have also been added to polymeric adhesives [219]. Therefore, it is envisaged that multifunctional tissue adhesives tailored for a particular tissue can be created by employing such nanocarriers.

The development of injectable adhesives that may crosslink in situ and produce nano topography to improve their adherence to surrounding tissues through van der Waals interaction or mechanical interlocking is one promising route for the area [220–222]. The subject of flexible and biodegradable electronics has dramatically benefited from recent advancements in nanofabrication methods. One possible path for the future is to build innovative tissue adhesives by fusing biodegradable electronics with polymeric tissue adhesives. Such intelligent adhesives can keep an eye on the injured site's healing process and the environment for any possible infections and inflammations. Overall, it is anticipated that tissue adhesives will become more popular due to the new features introduced to them, and they will be able to replace surgical staples and sutures in various surgeries [223, 224].

#### reconstruction

Stubinger et al. performed sinus floor elevations on 20 patients using nano-structured HA-based biomaterial and discovered that the material had outstanding tissue biocompatibility. They also reported that new trabecular bone grew within 6 months without the requirement for autogenous bone. According to the results of the histologic study, the nano-enhanced biomaterial functioned as a robust osteoconductive bone replacement with no indication of an ongoing noticeable foreign body response [106]. In dentistry and among maxillofacial surgeons, bioceramics are frequently utilized to restore missing teeth, close jaw deformities, perform mandibular reconstruction, and perform temporomandibular joint surgery [225]. The scientists remark that 3D porous nano-HA scaffolds containing bone-marrow seeds displayed cell adhesion, proliferation, and differentiation, providing promise for rebuilding bony defects [226]. In addition, Nanophase HA has been shown to have better osteointegration capabilities. Better implants can be created using nanophase HA, because it more closely resembles the nanostructure of natural bones and offers the potential for stronger osteointegration, more natural mechanical qualities, less immune reaction, and better control of cell responses. The consequences of craniofacial surgery are enormous [227].

### Surgical oncology

There has been a paradigm shift from tissue staining to tissue imaging with nanoparticles [228]. The primary and most efficient treatment option for cancer in humans is surgery [229, 230]. During surgery, the problems for the surgeons are the correct detection of cancerous tissues and the complete removal of the tumor with sufficient negative surgical margins [231, 232]. A unique characteristic of gold nanoparticles is known as the "Enhanced Permeability and Retention Effect" (EPR Effect) [233]. Gold NPs of a particular size assemble more in cancer cells than in healthy cells. Large surface areas and "Surface Plasmon Resonance" characteristics characterize gold nanoparticles [234]. Two different gold nanoparticle morphologies perform better in turning light into thermal energy. (1) Solid cylinders with a diameter of 10 nm or less are known as gold nanorods. The wavelength of light that a nanorod absorbs may be altered using various nanorod diameter and length combinations. (2) Nanospheres have a silica core with a gold covering [235, 236]. The wavelength of light that the nanosphere absorbs may change depending on changes made to the silica core's diameter and the thickness of the gold coating. Gold nanoparticles (AuNPs) have the following medical applications: spectroscopic cancer imaging, functionalized imaging agents for cancer detection, and prostate cancer treatment with fewer side effects than chemotherapy, and nanosized particles are particularly effective in evading the reticuloendothelial system are all examples of uses for these technologies [237–239]. A therapy approach known as theragnostic combines medicines with diagnostics [240, 241]. It is crucial to integrate cancer therapy, imaging, and diagnosis approaches. It is a helpful technique for mapping sentinel lymph nodes and evaluating tumor margins. They are employed to identify recurrent tumor cells or find micrometastases. They confidently support the surgeon's ability to remove the tumor with higher patient survival rates. Another benefit is the ability to identify nearby vital structures, lowering morbidity risk after surgery. Solid tumors collect nanoparticle contrast materials, which target active and passive processes and range in size from 10 to 100 nm. Their vast surface area makes it possible to combine several therapeutic and diagnostic substances for theragnostic [242, 243].

### The heart surgery

The identification and treatment of faulty cardiac valves and arterial plaque both benefit from the use of nanotechnology [244]. The structure of valves formed of collagen, a fibrous protein, is altered by nanomaterials, which are composed of tiny gold rods. As a result, off-the-shelf implanted tissue-engineered heart valves are needed (TEHVs). Microfabrication and 3-D hydrogel preparation techniques may soon realize a functioning and viable implanted TEHV assembly. In addition, more collagen is produced when growth-stimulating (steroid) medications are used to manufacture valves [22, 245].

To treat aneurysms and stop bleeding, nanomaterials with medications embedded in them are employed. Treatment for blocked arteries has improved thanks to nanotechnology. Known as micelles, nanoparticles are lipid-based molecules that have the shape of a spherical. Pectinase is released onto the plaque after being adhered to the surface. The nanoparticles, known as "nanoburrs," adhere to the desired region and release medication there for several days [22]. Creating highly functional artificial tissues makes it feasible to repair damaged heart tissues and other organs. They are strong and provide the tissues mended with them with enough support to perform at their best. Patches of heart tissue are created using hydrogel scaffolding strengthened by carbon nanotubes [246, 247].

### Vascular surgery

Vascular surgeons have previously created, tested, and used nanotechnology-based medical devices and therapies [248]. Drugs that have been modified using nanotechnology can be engineered to have extremely high binding specificity and can be directed toward antigens found in sick vascular tissue. Another illustration is nanofiber delivery systems, which may be utilized to boost the strength of specific medications. Utilizing nano delivery methods, nitric oxide and coenzyme Q10 (CoQ10) have been developed to prevent restenosis following angioplasty and reduce blood pressure [249]. As a result, the medications' systemic effects were reduced. For visualizing plaques and erosions in early vascular disease, nanoparticles can be employed. The ability of these particles to cross-link with specific fibrin peptides seen in sick plaques can be improved. With the aid of contrast chemicals for paramagnetic resonance imaging, the plaques may be scanned sooner, leading to early intervention [250].

The primary cause of transplant failure is thrombus development. Endothelial dysfunction is the cause. An ideal graft should have biomechanical characteristics similar to those of a healthy native vascular, the ability to withstand intimal hyperplasia and thrombo-resistant characteristics. Diamond-like carbon (DLC), used in brachytherapy for percutaneous coronary treatments, has unique electrical characteristics. More recently, coronary artery stents are also composed of DLC [22, 251].

### Ophthalmic surgery

One of the leading causes of cataracts and other visual illnesses is reactive oxygen species (ROS) [252]. The primary factor in cataract production is oxidation. Nanomaterials' high surface area to volume ratio is significant in treating many eye diseases [253]. Due to the eye's exposed position and ease of accessibility, nanotechnology has a wide variety of potential uses in several ocular disorders [253, 254].

- Measuring intraocular pressure and monitoring it continuously in glaucoma patients
- Care for newly formed arteries in choroidal theragnostic
- To avoid scarring after glaucoma surgery
- Gene therapy for the treatment of retinal degenerative illnesses
- Treatment of oxidative stress
- Defective (retinal) pigmented epithelial cells in age-related macular degeneration can be replaced by a scaffold to transfer stem cells.
- Regenerative nanomedicine and prosthetics in ocular surgery

Petrolatum and lanolin are components of artificial tears made of polymers. When used to treat dry eyes, they are connected to redness, itchiness, and discomfort in the eyes. They become a nanoscale-dispersed eye ointment when treated with medium-chain triglycerides as a liquid lipid and mixed with polyvinyl pyrrolidone solution (the ointment matrix particle size is about 100 nm) [255]. Noscale-dispersed eye ointment (NDEO) is safe for use in ophthalmic procedures and exhibits no cytotoxicity to human corneal epithelial cells. It brings back the natural morphology of the cornea and conjunctiva [256–258]. Patients with retinal degenerative illnesses do not have surgical scarring thanks to its better medication administration. Microemulsions, nanoparticles, nanosuspensions, dendrimers, liposomes, niosomes, and cyclodextrins are available as ocular drug delivery methods [22]. Future developments assisting millions of individuals in need include retinal nerve cell regeneration, nano retina, and nanotechnology-based eye implants [253, 259, 260].

### Thoracic surgery

Although nanotechnology has several uses in the realm of cancer, thoracic oncology is particularly well-positioned to gain from it, given the often dismal prognosis of thoracic [261]. With the help of medication or gene loading, endogenous or exogenous stimuli, endogenous or exogenous dyes, contrast or dye loading, or even a mix of these, NPs may be altered to give diagnostic and therapeutic capabilities customized to particular clinical applications [262]. Thoracic surgery, in particular, has the potential to benefit significantly from the use of nanotechnology, which is a young branch of medicine. With less systemic toxicity than conventional chemotherapeutics, treatment may be administered to tumors in a more focused manner by utilizing the unique qualities of many distinct nanometer-sized platforms [261]. Nanoparticles can help diagnose, pre-operative characterization, and intraoperative localization of thoracic tumors and associated lymphatics in addition to the packaged delivery of chemotherapy medications. There is a quickening increase of in vitro and in vivo investigations being carried out that give a better knowledge of possible toxicities and promise for more excellent clinical translation due to the growing interest in their therapeutic application [262]. The development of nanotheranostics, which promises to improve the treatment of thoracic malignancies through improved tumor targeting, controlled drug delivery, and therapeutic monitoring, is the result of focused research into the potential of nanotechnology to deliver both diagnostics and therapeutics [262].

In addition, NPs can be modified to contain different targeting moieties, such as ligands, aptamers, or antibodies, to target particular cancers actively. Pure pharmaceuticals, often between 100 and 1000 nm in size, are called nanocrystals. Several techniques, such as "bottom-up" (precipitation) or "top-down" (high-pressure homogenization) procedures, are used to prepare drugs. Due to their tiny size and amorphous form, nanocrystal drug delivery has a high saturation solubility [263, 264]. By enhancing the diagnosis, staging, therapy, and therapeutic monitoring of intra-thoracic malignancies, nanotechnology has the potential to enhance the area of thoracic surgery [262]. Scientists are learning how to address patient clinical demands by optimizing the material platforms to surpass constraints. Using nanocarrier contrast agents, imaging sensitivity and specificity are increased. While carefully analyzing the accompanying lymphatics, surgeons can more precisely pinpoint a lesion intra-operatively. Through stimuli-responsive platforms, drugs and inhibitory genetic material are being given in a "smart" way. Due to all of these developments, "nano theranostics" multifunctional NPs have emerged to address the difficulties of clinical application [262, 265–267].

### Catheters for minimally invasive surgery

Catheters are tiny tubes placed into the human cavity to inject, drain, or maintain the clearness of a channel. The development of thrombus on the outside of catheters is one of their problems. To improve the strength and flexibility of catheters used in minimally invasive surgery and lessen their thrombogenic impact, nanomaterials, such as carbon nanotubes, have been effectively incorporated. The nucleation function of carbon nanotubes has presumably improved electrostatic characteristics and dense surface topology, which have contributed to the antithrombotic properties [268]. In addition, silver nanoparticles can coat catheters to provide antibacterial qualities and stop the growth of surface biofilm [269]. The potential of thrombus development on the surface of intravascular catheters is the most feared complication in interventional cardiology. Recent developments in minimally invasive surgery can be attributed to catheters built of nanotube-based polymer reinforced with multi-walled carbon NTs [22]. The carbon nanotubes' nucleation function lowers thrombogenicity by modifying electrostatic characteristics and dense surface alterations. Enhanced mechanical qualities, quicker return to the original form and improved handling properties, high fracture resistance, and the ability to prevent and treat biofilm-associated infections on medical devices are all benefits of nanotechnology-based products [251].

## Other applications

A significant source of nosocomial infections is the operation room. The absence of infection control procedures is one of the main contributing reasons for operating room infections. Despite the numerous advantages of surgery, which in most cases saves patients' lives, its drawbacks, including postoperative infections, should be taken into account, and necessary steps should be taken to lessen these infections. One of the primary causes of nosocomial infections while utilizing medical tools and equipment to diagnose and treat patients is bacterial contamination of those tools and equipment [270, 271]. Unfortunately, the risk of infectious illnesses puts human life in danger by making microorganisms more resistant to harsher drugs. Metal oxide nanoparticles are a viable alternative to antibiotics for treating bacterial infections. Today, several techniques have been employed to assist people in reducing their use of these antibiotics. Bacterial inhibition by nanoparticles prevents the development of antibiotic resistance [272, 273].

The mechanism of action of silver nanoparticles is that cellular respiration in the presence of various nanosilver concentrations in various bacterial groups exhibits a similar pattern, which is a gradual decrease in cell respiration as the concentration of nanosilver increases rather than at MIC for the bacteria [274]. The lowest respiration rate for each bacteria is reached. Due to their greater surface area per unit volume, metal nanoparticles, including those made of copper, titanium, magnesium, zinc, gold, and silver, have considerable antibacterial potential [275, 276]. The most efficient antibacterial agent against bacteria, viruses, and other eukaryotic microorganisms has been demonstrated to be silver nanoparticles. The development of nanosilver goods has focused chiefly on antibacterial materials, which have been transformed by nanotechnology and have several benefits over chemicals. A range of physical, chemical, and biological processes, including charge–discharge, chemical reduction, sonochemistry, irradiation, cytochemical synthesis (based on green chemistry), and talc techniques, are used to convert silver to nanoparticles in a fine-grained manner [277–279]. Silver has antibacterial, antiviral, and antifungal characteristics. It also has no allergies, strong performance at low dosages, good antimicrobial stability after numerous washes, and the capacity to decrease smells and preserve equilibrium. Fungicides with a short half-life are efficient against widespread fungi, such as Aspergillus, Candida, and Saccharomyces. AIDS and influenza viruses may be prevented from replicating by silver nanoparticles ranging in size from 5 to 28 nm [280–283]. Zinc oxide nanoparticles indicated many applications [284, 285]. A valuable role in wound healing has also been played by zinc oxide (ZnO) and silver. The United States Food and Drug Administration has classified ZnO as safe. Since they are smaller and have a higher surface-to-volume ratio than larger particles, ZnO nanoparticles interact with bacteria more effectively than larger particles. Zinc oxide nanoparticles (ZnO NPs) have been proposed by researchers as an improved disinfectant and antibacterial agent for microbes [286, 287].

### Stem cell-incorporated nano scaffolds

Creating nano scaffolds that mirror the physical properties of human skin is a fascinating method for using MSCs [193, 288]. The use of polymeric nanofibers in tissue regeneration has also increased recently [289]. These nanofibers may mimic natural tissue and are biomimetic, which makes them the perfect material for a stem cell niche. The capacity of BM-MSCs to adhere to a collagen/PLGA nanofiber scaffold to achieve a quicker closure of cutaneous wounds was observed. One of the great and often utilized uses for nanotechnology is wound care. With promising outcomes, monotherapies target several stages of wound healing [290–293]. Chronic wounds are a tremendous burden, but there are several authorized treatments. Bioengineered human dermal replacements, recombinant platelet-derived growth factor (rhPDGF), and skin equivalent are appropriate for treating chronic wounds. Smart biomaterials such as dermal replacements and human skin substitutes encourage a healthy healing process by interacting with the environment around the wound. The advantageous effects of PDGF on wound healing include the stimulation of neutrophils, macrophages, fibroblasts, and smooth muscle cell proliferation [294–296].

### Nano-surgery—laser surgery

Nanoscale laser surgery is a novel idea created due to recent advances in nanotechnology. Femtosecond laser pulses, one-millionth of a billionth of a second, can be used to control cellular structures at the nanoscale. For example, a single strand within a cell may be selectively severed using a Femto laser. This implies that a cell's organelles might be removed without affecting the remainder of the cell. As a result, it could offer the best instrument for ophthalmological surgery [297].

On the other hand, nano-cryosurgery offers a fresh take on an established idea. The idea is to load certain nanoparticles into and around cancer tissue. Due to their great cooling conductivity, they will quickly freeze at lower temperatures. The induction of cancer cell death considerably enhanced the effectiveness of the cure by freezing action. Nano-cryosurgery technology is a potential option for tumor management in the future despite the challenges that still need to be addressed before it can be employed in clinics [298].

### Hemostasis

To achieve hemostasis in a matter of seconds, a self-assembling peptide that creates a nanofiber barrier has been used. The peptides self-assemble into a nanoscale protective barrier gel when applied to open wounds, sealing the wound and stopping bleeding. In addition, the non-toxic gel is broken down into molecules that cells may employ as the foundation for tissue rebuilding once the wound has healed. All types of surgery will be affected by this process in significant ways [299].

### Nerve repair and regeneration

Both academics studying nanotechnology and plastic surgeons are particularly interested in nerve regeneration [300, 301]. Nerve grafting, typically from an autologous donor source, is commonly necessary for traumatic nerve injuries that leave gaps in the nerves greater than 5 mm. New methods of peripheral nerve restoration have been developed using nanoscale manufacturing techniques to minimize the morbidity of autologous nerve grafting. To help regenerate nerves, tubular and porous nanostructured conduits have been created utilizing various natural materials. These conduits have then been filled with different biomaterials or cell types, such as neural stem cells, Schwann cells, or embryonic stem cells [302].

### Nanorobots

Recently, robots have been used to do routine surgical procedures, and with the aid of nanobiotechnology, another aspect of robotics has been established [303]. This is also referred to as nanobots or nanorobots. These tiny robots are so small that they may be inserted into the body through the vascular system or by catheters. With the assistance of the surgeon's external direction and supervision, they can perform precise intracellular surgery that is impossible to perform with the human hand. Due to its strength and inertness, carbon atoms arranged in a diamondoid form are likely to be used to build nano-robot exteriors. The likelihood of the body's immune system being activated will be reduced by highly smooth surfaces, allowing the nanorobots to operate without hindrance. Depending on the purpose, the nanorobot may use oxygen, glucose, or other natural body sugars as a source of propulsion [304, 305]. It may also include other biochemical or molecular components. An autonomous on-site surgeon within the human body might be created by a human surgeon using a surgical nanobot [306]. An onboard computer may conduct several tasks, including pathology detection, diagnosis, and removal or repair of the lesion by nanomanipulation, all while staying in touch with the supervising surgeon through coded ultrasound signals. The use of nanoparticles during surgery already makes malignancies visible, allowing doctors to entirely remove them or even visually check for metastases throughout the body. To identify and cure cancer in its earliest stages, nanoparticle-based local medication delivery will soon make it possible to diagnose and treat cancer more cell-to-cell [307, 308].

Nanobot aims to challenge the fundamental paradigm of modern medicine using in-body sensors that look for and kill germs before the patient has any symptoms [309]. Applications of nanorobotics include the elimination of cancerous cells and diseased plaques, the healing of damaged tissues, molecular monitoring of bodily function, and the enhancement of human health and functioning. Nanorobots can be made of biochips, microorganisms, positional nano assemblies, or Nubots (nucleic acid robots). Its strength comes from the diamondoid structural shape. Miniature robots are created to be inserted into the body's circulatory system. They can use catheters to do simple surgical operations as well. The robot is powered by movement, force, or a signal generated at the cellular level. Through external direction and supervision, precise intracellular surgery may be carried out. Clinical uses for nanorobots include illness monitoring, disease diagnosis, disease treatment, and targeted medication administration [16, 310–312].

### Nanotechnology in imaging

Using conventional imaging methods, diseased tissue is found, and contrast chemicals are injected to locate the disease. Nanotechnology refinement blends contrast chemicals and imaging techniques to identify illness even at the single-cell level [313]. Nanotechnology makes multifunctional imaging programs feasible because of the nanoparticles' large surface area. Both molecular magnetic resonance imaging (mMRI) and radionuclide-based molecular imaging techniques are quantitative and sensitive, although their resolution is often modest (> 1mm). Nanoparticle-based multimodal imaging provides excellent accuracy rates and improved intrinsic and extrinsic resolution. Magnetic iron oxide indicated various applications [314]. Imaging labels such as superparamagnetic iron oxide (SPIO) nanoparticles are used for cellular imaging and medication administration [315, 316]. Nuclear imaging of tumor angiogenesis and detecting atherosclerosis and cancer employ quantum dots and microbubbles. The benefits include creating a stable colloidal solution and achieving magnetization in a magnetic field. In terms of magnetic targeting, local hyperthermic effects, and visualization using magnetic resonance imaging, SPIO is exceptional, because they function as carrier vehicles. SPIOs are standard diagnostic instruments that integrate illness diagnosis, treatment, and follow-up. SPIO crystals are stimulated by an external alternating magnetic field, which results in local hyperthermia-based treatments. Infiltration of SPIO-labeled macrophages serves as a substitute marker for stem cell viability [317, 318].

### Nanotechnology in dermatology

Dermatology has already benefited greatly from advances in nanotechnology [319]. A cosmetics firm is reportedly the sixth-largest nanotechnology patent holder. This tendency will persist as the market for cosmetic skin care continues to expand quickly [320]. Research is being done on the possibility of transdermal medication delivery utilizing nanocarriers. Injectables will soon be replaced by a new transdermal Botox and filler industry once they are proven secure and reliable. The use of nanotechnology will enable this [321]. Recently, Pornpattananangkul et al. [322] created functionalized, tunable, acid-responsive liposomes that may be employed as cutaneous medication nanocarriers to treat staph infections and acne vulgaris. Nanogold particles are placed on these liposome carriers to stabilize them and stop them from prematurely fusing and growing too massive to pass through the epidermal barrier. The gold nanoparticles penetrate the skin's acidic environment, separate from the liposomes, merge with the cells, and release their payloads.

In a bleaching regimen on 84 individuals with 88 hyperpigmented lesions, nano-all-trans-retinoic acid (nano-atRA) gel was shown to reduce hyperpigmentation. Like the traditional atRA gel, it caused irritating dermatitis with less erythema. However, in comparison with atRA, nano-atRA was more stable and had a long storage life. In addition, it showed improved stratum corneum penetration and the anticipated delayed release feature [322, 323].

### Diagnostic applications

The use of nanotechnology in cancer detection relies on binding complexes made of nanoparticles and ligands to specific genetic alterations that enable fine-grained cellular imaging [324, 325]. When a contrast agent is added to these complexes, the tumor cells that express the particular mutation may be seen. The p15 gene, a tumor marker mutation frequently linked to lung metastasis in osteosarcoma, has been used to study this method. The use of this technique could make it possible to detect a malignancy's metastatic potential early on. Chemotherapy can be started before clinical signs show up when combined with medication delivery using nanotechnology, reducing patient morbidity. In addition, the assessment of the effectiveness of treatment for cancer may be aided by the detection of nanomaterials employing fluorescent probes. This approach may provide more accurate tumor residual measurement than histology examination following tumor removal [326–328].

### Femtosecond laser neurosurgery

The likelihood of tissue injury while using a traditional LASER is significant, since it produces heat before cutting. By performing an exceedingly accurate surgery, ultra-short pulse lasers are utilized to sever nanosized cell structures, such as nerve cells. " Nano scissors" create low-energy, femtosecond near-infrared laser pulses without endangering the healthy tissues around them. The thermal damage caused by the brief and low-energy laser pulses is modest. Reduced mechanical effects such as plasma extension and shock waves are to blame for this. In addition, there is no buildup of heat and no thermal harm to healthy surrounding tissues. Within 24 h, 50% of the severed axons showed signs of regeneration. In dermatology, ophthalmology, and corneal refractive surgery, femtosecond laser systems are used [329, 330].

### Manufacture of surgical implants and tissue engineering products

All surgical specialties have significantly benefited from the use of nanotechnology in the production of surgical implants and tissue engineering products, which also advances imaging technology, drug delivery systems, and scaffolding by improving the interaction of materials and cells, which has been successful in wound healing [22]. Significant benefits of surgical blades built of diamond monolayers include. Diamond reduces the penetrating force in tissues, since it has a low coefficient of friction. Its physical adherence to materials or tissues is minimal. Blade with plasma polishing is offered as Diamaze PSD—Plasma Sharpened Diamond. With a surface roughness reduction of 20–40 nm, the coating thickness has been lowered from 5–25 m to 0.5 m. They are perfect for precision surgery in the disciplines of plastic, ophthalmology, and neurosurgery. Sandvik Bio-line nanotechnology (1RK91 ™) produces medical wires with extremely high tensile and hardness characteristics [22].

#### Nanocoated surgical blades

One may see a surgeon able to alter and monitor individual cells by creating a new set of tools on the nanoscale. For example, the neurosurgical elements might be highly advantageous, and the patient will also profit from the lessened trauma of even tiny incisions. The processing of microstructured hard metal with diamond coating can significantly improve the performance of surgical blades. Diamond nanolayers' minimal physical adherence to substances or tissues and chemical and biological inertness are significant benefits in this application. Diamond also has a low coefficient of friction, which lowers the required piercing force [331]. New manufacturing techniques have made it possible to produce surgical blades with cutting-edge diameters between 5 nm and 1 m. For use in ophthalmology, neurosurgery, and minimally invasive surgery, diamond scalpels with cutting edges that are only a few atoms thick (about 3 nm) have been developed. The scalpel blade is around one-thousandth the breadth of a metal blade [17, 98].

#### Suture needles and sutures

A novel method for preventing surgical infections uses silver nano-coating on sutures [332]. To efficiently control bleeding and mend injured tissues, aqueous nanoparticle solutions containing Stöber (Mesoporous) silica or iron oxide are employed for nano bridging [333, 334]. For the treatment of postoperative pain, electro-spinning drug-eluting sutures with or without bupivacaine have been created [335]. Sutures and medications such as aceclofenac or insulin release the medication gradually over time. Insulin promotes wound healing by enhancing cell migration, whereas aceclofenac inhibits epidermal hyperplasia and skin irritation [336, 337]. Antibiotic-eluting sutures reduce the risk of infection while achieving good healing qualities [338]. For eye surgery, absorbable antibiotic-eluting sutures containing poly(L-lactide), PEG, and levofloxacin are utilized successfully [338]. Using atomic force microscopy, nanoneedles are made from pyramidal silicon AFM tips using focused ion beam etching, and carbon tubes are affixed to the tips of the needles. Potentially, a nano surgeon might operate on a single cell without endangering it [339, 340].

#### Breast implants and capsular contracture

According to Barr et al., micro- and nano-topographies impact fibroblasts and the long-term bio integration of devices, such as breast implants. They cite research showing that cell filopodia can detect "nano islands down to a size of 10 nm." The consequence is that the nanoscale design could affect how the body reacts to a breast implant and how capsular contracture eventually develops. In their investigation, Barr et al. looked at the surface architecture of the shells of five breast implant materials that have been given UK approval. They discovered a significant amount of variation in surface properties. In particular, they discovered that smooth implants had a directional "shallow, regular, 5-km period rippling pattern" that is considered to be caused by how the implants are made. This surface design is hypothesized to stimulate fibroblasts to produce capsules, which accounts for the smooth implants' increased risk of capsular contracture. Contrarily, it is believed that Biocell and Siltex surfaces with random surface characteristics 100 to 200 km deep secure implants in place and lessen contracture. In particular, they discovered that smooth implants had a directional "shallow, regular, 5-km period rippling pattern" that is considered to be caused by how the implants are made. This surface design is hypothesized to stimulate fibroblasts to produce capsules, which accounts for the smooth implants' increased risk of capsular contracture. Contrarily, it is believed that Biocell and Siltex surfaces with random surface characteristics 100 to 200 km deep secure implants in place and lessen contracture [341].

Nanoscale manufacturing advancements might increase the strength of breast implants. The silicone rubber nanocomposite used to create silicone breast implants is cross-linked and strengthened. Due to its fragility, silicone rubber must be strengthened, most frequently with nanoscale SiO_2_. The shell looks to be transparent when this is done. Finally, capsular contracture continues to be one of the most common long-term problems of long-term breast implant installation despite advances and breakthroughs in implant manufacture. Surface modification of implants using antifibrotic medications, such as halofuginone, has reduced capsule formation in a rat model. Estimates place the yearly incidence of Grade 3–4 breast implant capsular contracture (i.e., needing surgical intervention) at 9–27%, or 22,000–44,000 women. These challenging operations often cost around $7500. Therefore, creating a breast implant with a lower capsular contracture profile might significantly impact how this illness develops [95, 342, 343].

## The ethical and safety aspects of nanotechnology

During the last two decades, nanomaterials research and manufacture have sparked heated public discussions regarding potential toxicological, health, and environmental consequences. According to recent studies, toxic nanomaterials have been found in various settings, including air, water, soil, plants, and human bodies [344–348]. These invisible compounds can float for days or weeks in air and water, creating significant dangers during manufacturing, shipping, handling, consumption, trash disposal, and recycling operations. Asthma, lung and liver cancer, bronchitis, Parkinson's and Alzheimer's disease, heart disease, Crohn's disease, colon cancer, and birth abnormalities can all be caused by nanomaterial exposure [349, 350]. The toxicity of nanomaterials can be regulated by their surface attributes, which include potential, shape, charge, reactivity, particle size, and surface area [344, 351]. These characteristics can influence their capacity to interact with the human body, stay inactive, or respond to it. According to research, nanoparticles having a larger surface area are more hazardous to human and animal cells [352]. Electrostatic interactions between nanoparticles and cells can increase their penetration rate in sensitive organs and tissues, such as the central nervous system, brain, bone marrow, spleen, lymph nodes, and heart. This raises concerns regarding nanomaterial toxicity and potential damage to the human body [353–356].

Nanoparticles agglomerate to form bigger clusters at lower potentials, which are less hazardous than individual particles. Smaller particles can enter sensitive regions more quickly, producing long-term health problems [352, 357]. The synthesis of novel nanomaterials using various approaches generates worldwide uncertainty and concerns. The study of nanotechnology and nanomaterials, known as nano ethics, gives principles for training, restriction, and restraint in the usage of these nanoparticles. It is critical to decrease risk factors and public concerns before producing and using nanomaterials in consumer products [352, 358, 359].

The lack of awareness about the dangers and liabilities that nanomaterials may cause to workers is at the root of ethical concerns concerning nanotechnology in the workplace. This necessitates a preliminary assessment of potential hazards and risks. Workers' autonomy may only be exercised if the methods for danger identification and evaluation are clear and understood. Employers must conform to autonomy, beneficence, nonmaleficence, fairness, privacy, and respect for humans by correctly depicting dangers, being precautionary, communicating with employees, and limiting risks to appear reasonable and acceptable to them [360]. Adopting new technologies by the general public is essential to their development. The general public should be informed of the advantages, possible concerns, and necessary precautions associated with using nanotechnology. Public debate should be fostered to enable individuals to create their own opinions. Scientists should play a key role in educating the public about the ideas and uses of nanotechnology [361, 362].

## The future of nanotechnology

Nanotechnology has enormous potential in the world of medicine [20]. Researchers are investigating the creation of nanoscale drug delivery devices that may precisely target-specific cells or tissues, boosting therapy efficacy while reducing adverse effects [363–365]. Nanomedicine has the potential to offer customized medicine by providing individualized therapies based on a person's genetic composition [366–368]. Furthermore, nano sensors have the potential to transform diagnostics by allowing real-time monitoring of health problems at the molecular level [369–371]. Nanotechnology can potentially play a critical role in tackling energy and environmental issues [372]. It might aid in creating more efficient solar cells, sophisticated energy storage devices, and lightweight, high-performance energy conversion and storage materials [373–376]. Nanomaterials may enable more efficient water filtration and pollution remediation technologies [377]. We might make tremendous advances in renewable energy and environmental practices using nanotechnology [372]. Nanotechnology is already significantly influencing the electronics sector, resulting in smaller, quicker, and more powerful gadgets [378, 379]. Nanoscale materials and components may improve the performance of electronic devices in the future, allowing new functionality and applications [380, 381]. Researchers are investigating tiny transistors, quantum computing, and nanophotonics to push the frontiers of computer and communication technology [381–384]. Materials science and manufacturing processes might be transformed by nanotechnology. Scientists may design materials with increased qualities such as strength, conductivity, and flexibility by altering the arrangement of atoms and molecules at the nanoscale. This opens the door to novel lightweight materials, enhanced coatings, and nanocomposites with superior mechanical, thermal, and electrical properties [385–388].

Nanotechnology may potentially allow more accurate and efficient production procedures. As nanotechnology advances, examining the possible environmental and health consequences is critical [389, 390]. To ensure the safe development and deployment of nanotechnology, researchers are actively investigating the impact of nanoparticles on human health and the environment [391]. Strong laws and standards will be essential in minimizing possible hazards and reaping the advantages of nanotechnology [392, 393]. Several research topics, such as nanotoxicology [394], nanofabrication [395], quantum nanoscience [396], and bionanotechnology [397], are crucial for the future growth of nanotechnology. These are only a few potential elements of nanotechnology's future. We may expect further breakthroughs and applications in numerous disciplines as research advances and discoveries are discovered, enhancing our lives and influencing the world.

## Conclusion

Nanotechnology is the atomic, molecular, and supramolecular manipulation of matter. Molecular biology, health and medicine, materials, electronics, transportation, drugs and drug delivery, chemical sensing, space exploration, energy, environment, sensors, diagnostics, microfabrication, organic chemistry, and biomaterials are just a few of the many scientific fields, where it has a wide range of applications. Innovations in medicine delivery, fabric design, material reactivity and strength, and molecular production are all part of nanotechnology. The use of nanotechnology in practically all surgical disciplines has transformed how numerous medical and surgical diseases are treated. Nanotechnology is used in surgical specialties to produce surgical tools, suture materials, imaging, targeted medication therapy, visualization techniques, and wound healing approaches, among other clinically valuable uses. Nanotechnology is widely used in the treatment of burn wounds and scars. Technology is essential for patients' functional recovery in preventing, diagnosing, and treating many orthopedic disorders. With nanotechnology, clinical trials, research, and the production of safe medical equipment are all enhanced. Researchers are working very hard to discover applications for nanotechnology in operating rooms. The surgical tools and hospital equipment sector is where these materials are most crucially used to prevent and control infection. By learning more about the circumstances of manufacturing and the structure of nanomaterials, we can move them from the nook of research labs closer to patients' beds. Therefore, there is much promise for the therapeutic use of these novel materials. Although the therapeutic usage of this nano-corn has reportedly seen some success, increased utilization and adequate understanding of nanomaterials demand more developments in the creation and design of new materials. Our goal in doing this study was to compile essential data in researching the use of nanotechnology in operating rooms. All instances of nanotechnology use in surgical procedures were classified in this study based on foundation, application, and mechanism. It was reported on the potential and restrictions of operating room nanotechnology. Researchers may find the material from this page valuable in designing and developing chemical disinfectants, instruments, equipment, and treatments for specific illnesses. A fascinating field of medicine called nanotechnology is quickly moving from in vitro and in vivo testing to clinical settings. Nanoparticles can deliver medicines at higher local concentrations with less systemic toxicity thanks to focused drug delivery. There are now many different material platforms, each with unique benefits and drawbacks. Nanoparticles have great potential for the future of medicine and surgery with a more excellent investigation into their mechanisms of action and safety profiles. Nanotechnology developments have affected medication delivery methods, tissue engineering, and implant design. The use of these novel technologies will grow dramatically as our knowledge of physiology at the nanoscale improves. To transition from a "foreign body response" to "known bidirectional body interaction," additive surface modification methods that produce a controlled nanotexture on implant surfaces have demonstrated promising outcomes in terms of bacterial growth and guided tissue integration.

Designing implants that can give satisfying solutions by minimizing clinical consequences, in the long run, requires a thorough understanding of how surface features affect inflammatory cell response. Our capacity to comprehend biological complexities and address physical and medical issues by creating deft biomimetic approaches is being revolutionized by advances in nanobiotechnology. Nanobiomaterials have a promising future in orthopedic applications, according to preliminary studies, but substantial progress must yet be made before they can be used in patients. Implanted interfaces, tissue engineering, and medicines have long been the main study fields. These diverse fields now have more capabilities thanks to nanotechnology. By deploying surgical instruments, such as nanoneedles and nanosurgical blades, improving diagnostic imaging, and using precisely targeted drug delivery systems, nanotechnology will simplify surgeons' lives. In addition, enabling more precise treatment planning and improving treatment plan execution will aid in lowering problems.

All areas of medicine and health care have been enhanced by nanotechnology. Patient care has improved due to developments in medication and gene delivery, biomedical imaging, and diagnostic biosensors. It is vital in many surgical disciplines but essential for cancer detection, imaging, and treatment. Future development of complex and inventive hybrid technologies is possible, given the scope of their applications. Single-walled carbon nanotubes make biomolecule delivery transporters that are highly effective. It is feasible to manipulate genetic material with nanotechnology and find new biological components. The best treatments for wound healing and burn injury care help patients feel less pain and heal their wounds. The paradigm of wound care will evolve as smart bandages emerge in the near future. The potential uses of medicine, regenerative medicine, stem cell research, and nutraceuticals will expand with more studies. All discoveries, breakthroughs, and helpful advancements will eventually benefit humanity and alleviate human suffering.

## Data Availability

By ordering from the corresponding author.
